# Supplementary material for: High-density linkage maps and chromosome level genome assemblies unveil direction and frequency of extensive structural rearrangements in wood white butterflies (Leptidea spp.)
Source: Chromosome Res. 2023 Jan 20;31(1):2. doi: 10.1007/s10577-023-09713-z (PMC9859909; doi:10.1007/s10577-023-09713-z)
Supplement: Supplementary file 1 — (PDF 15842 kb) [file 10577_2023_9713_MOESM1_ESM.pdf]

Supplementary material

Supplementary figures

a)

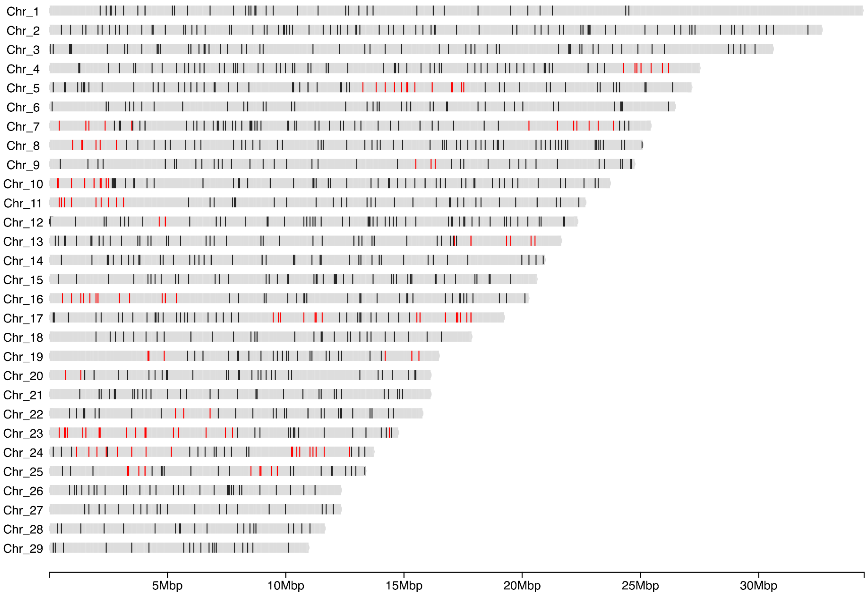

b)

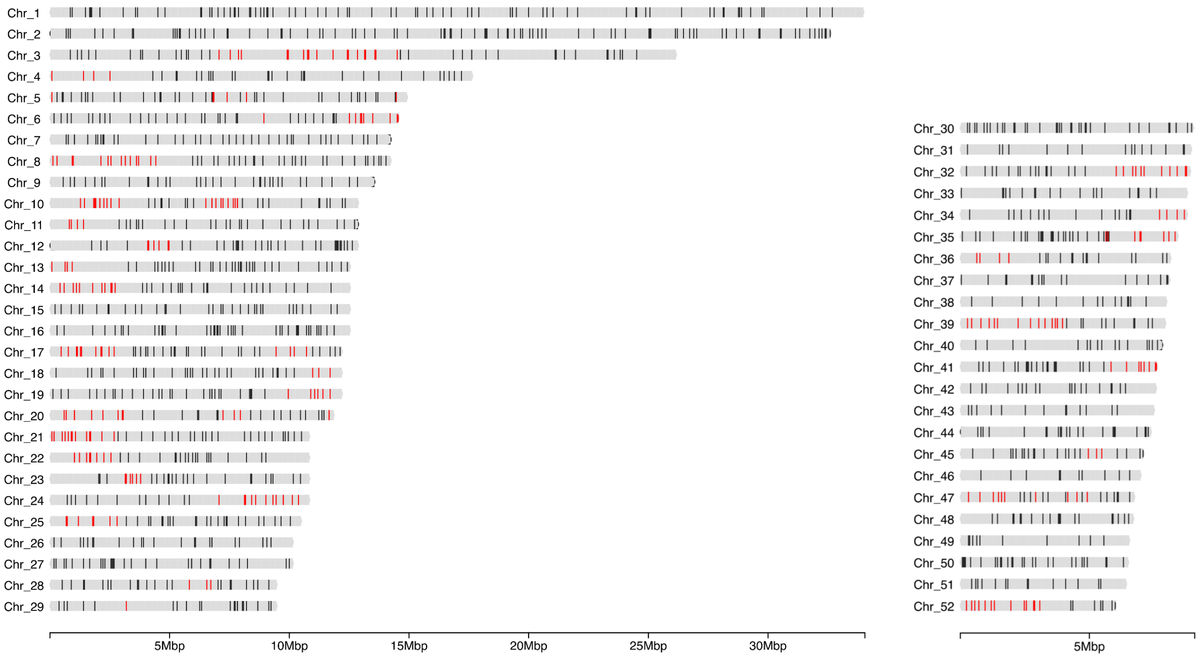

Supplementary figure 1. Linkage maps based on pedigrees for a) the Swedish, and b) the Catalan populations of L. sinapis. Vertical bars on each chromosome represent the position of linkage informative markers ordered according to the physical map (genome assembly). The x-axis represents the physical position of each marker in megabases (Mb). Red colour represents markers where the marker order in the linkage map was found to deviate from the order in the preliminary physical assembly.

A

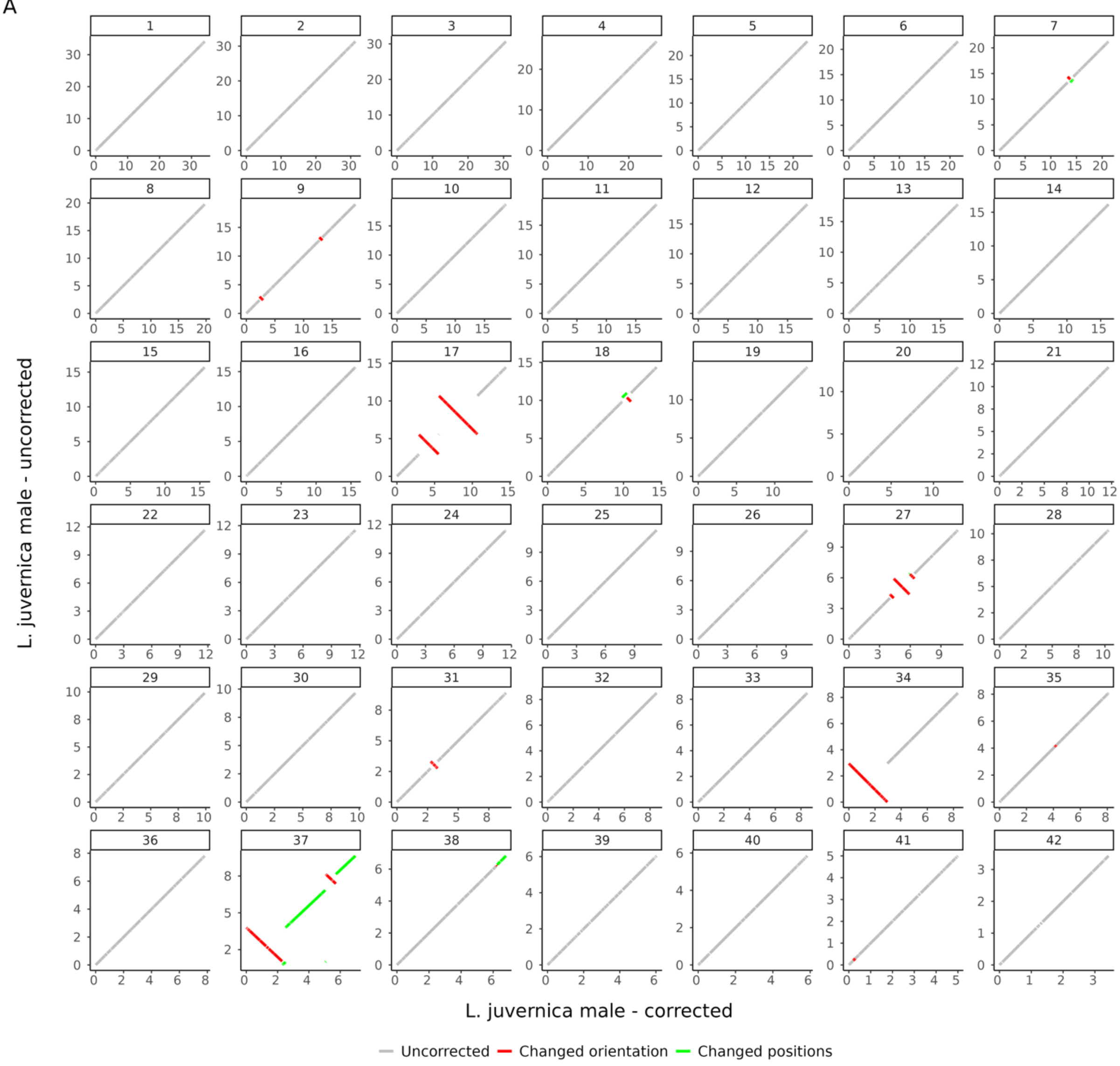
L. juvernica male - uncorrected
L. juvernica male - corrected
Uncorrected
Changed orientation
Changed positions

B

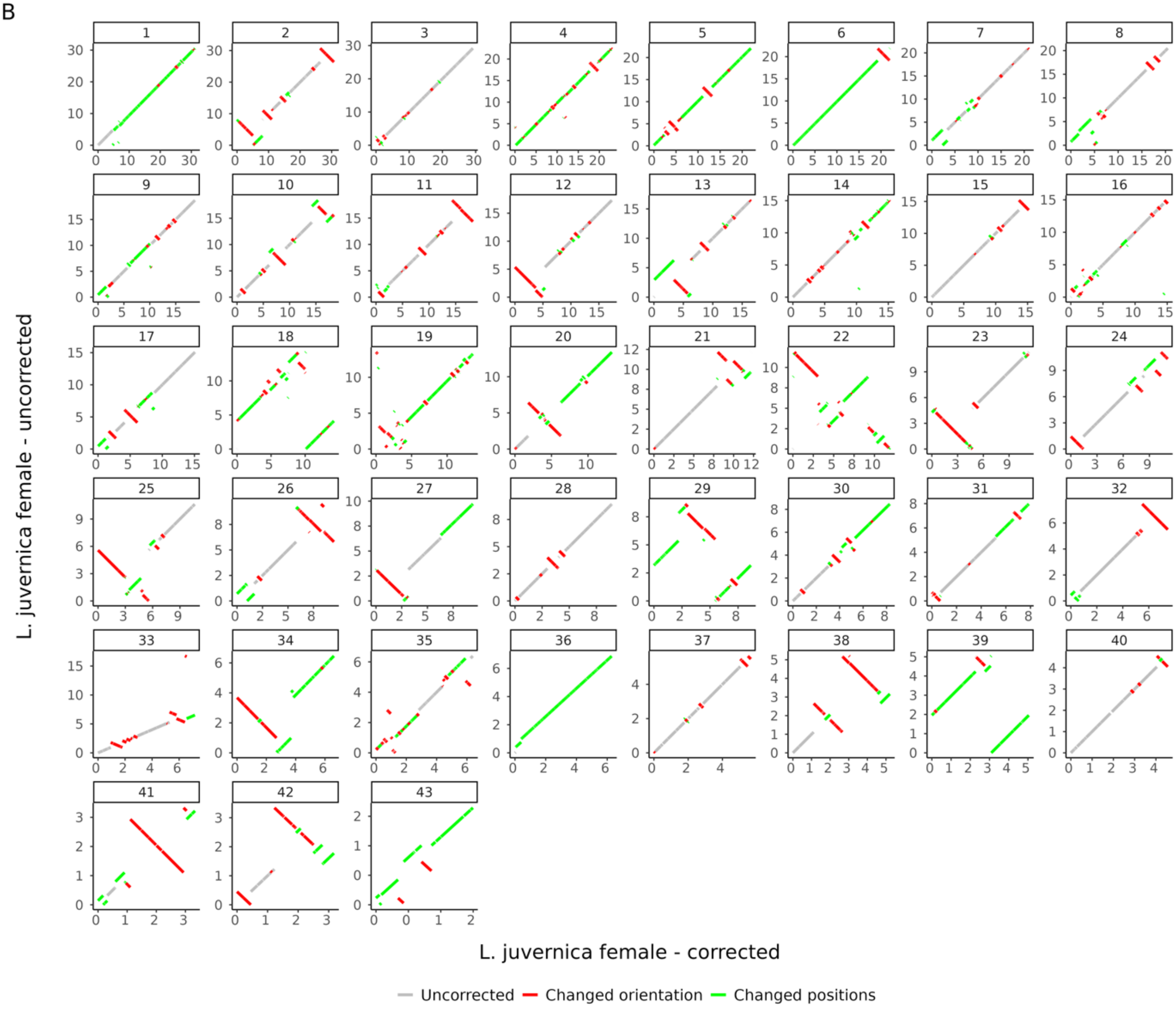

L. juvernica female - uncorrected
L. juvernica female - corrected
Uncorrected
Changed orientation
Changed positions

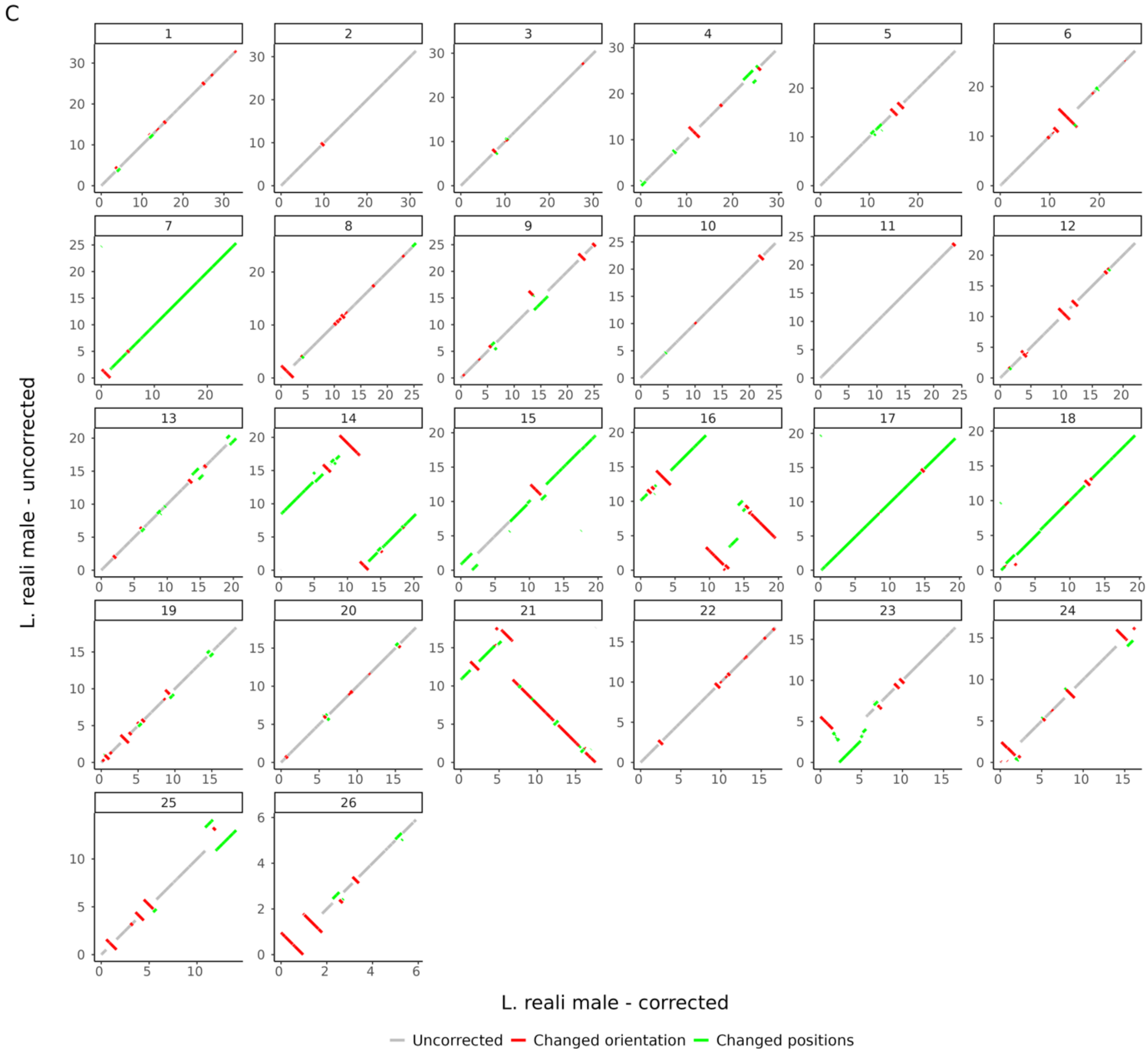
C
L. reali male - uncorrected
L. reali male - corrected
Uncorrected
Changed orientation
Changed positions

D

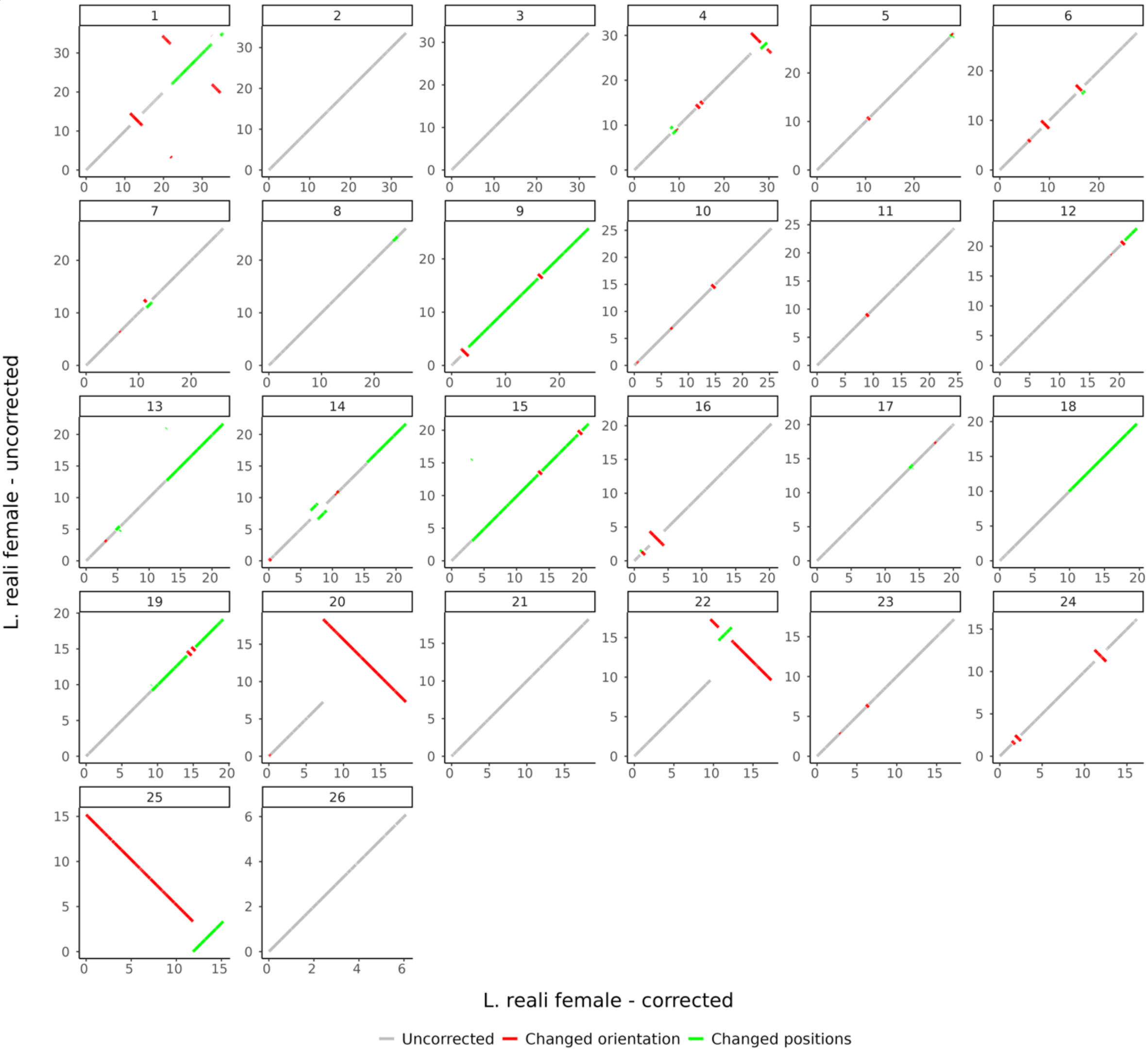

L. reali female - uncorrected
L. reali female - corrected
Uncorrected
Changed orientation
Changed positions

E

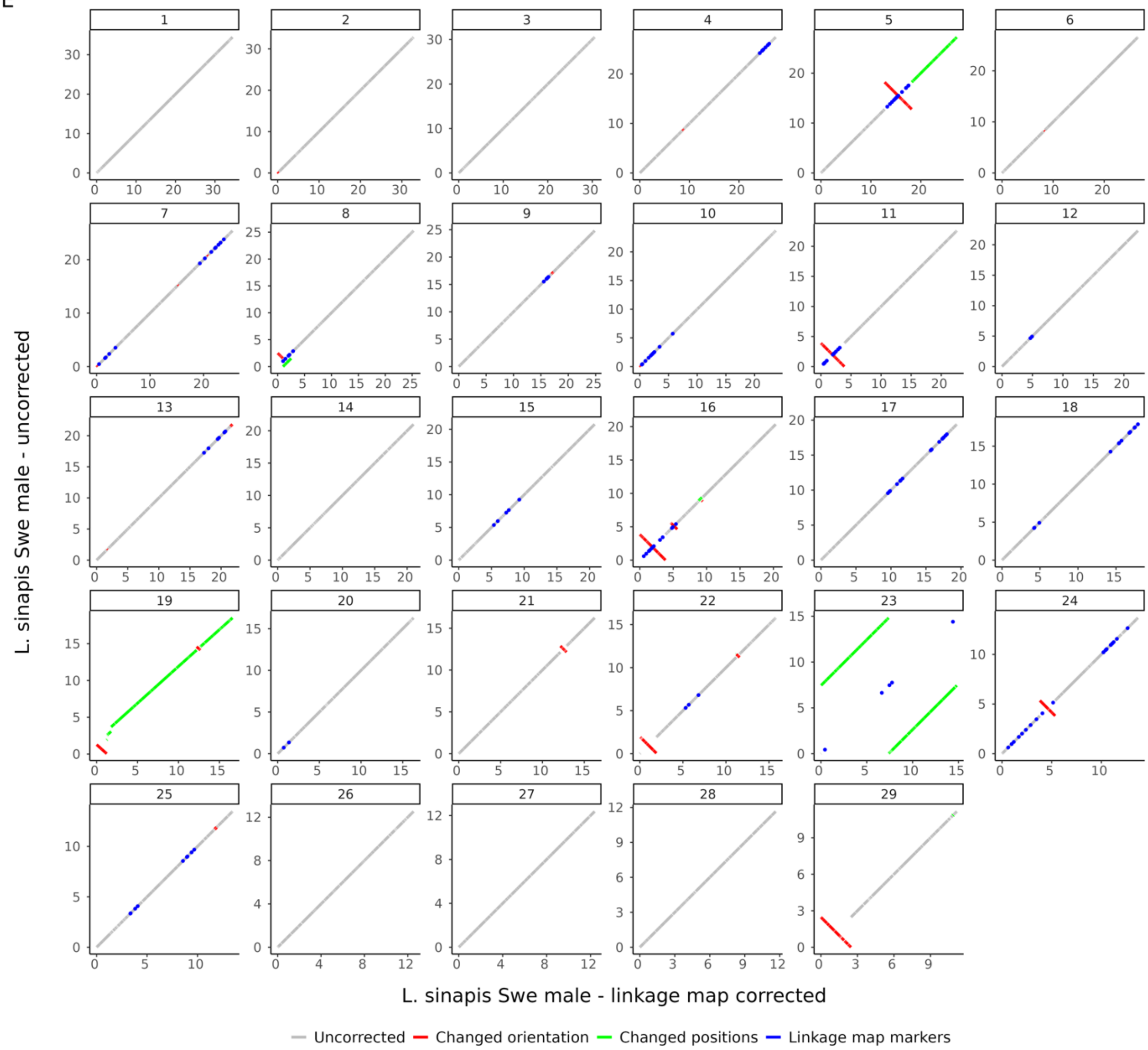

L. sinapis Swe male - uncorrected
L. sinapis Swe male - linkage map corrected
Uncorrected
Changed orientation
Changed positions
Linkage map markers

F

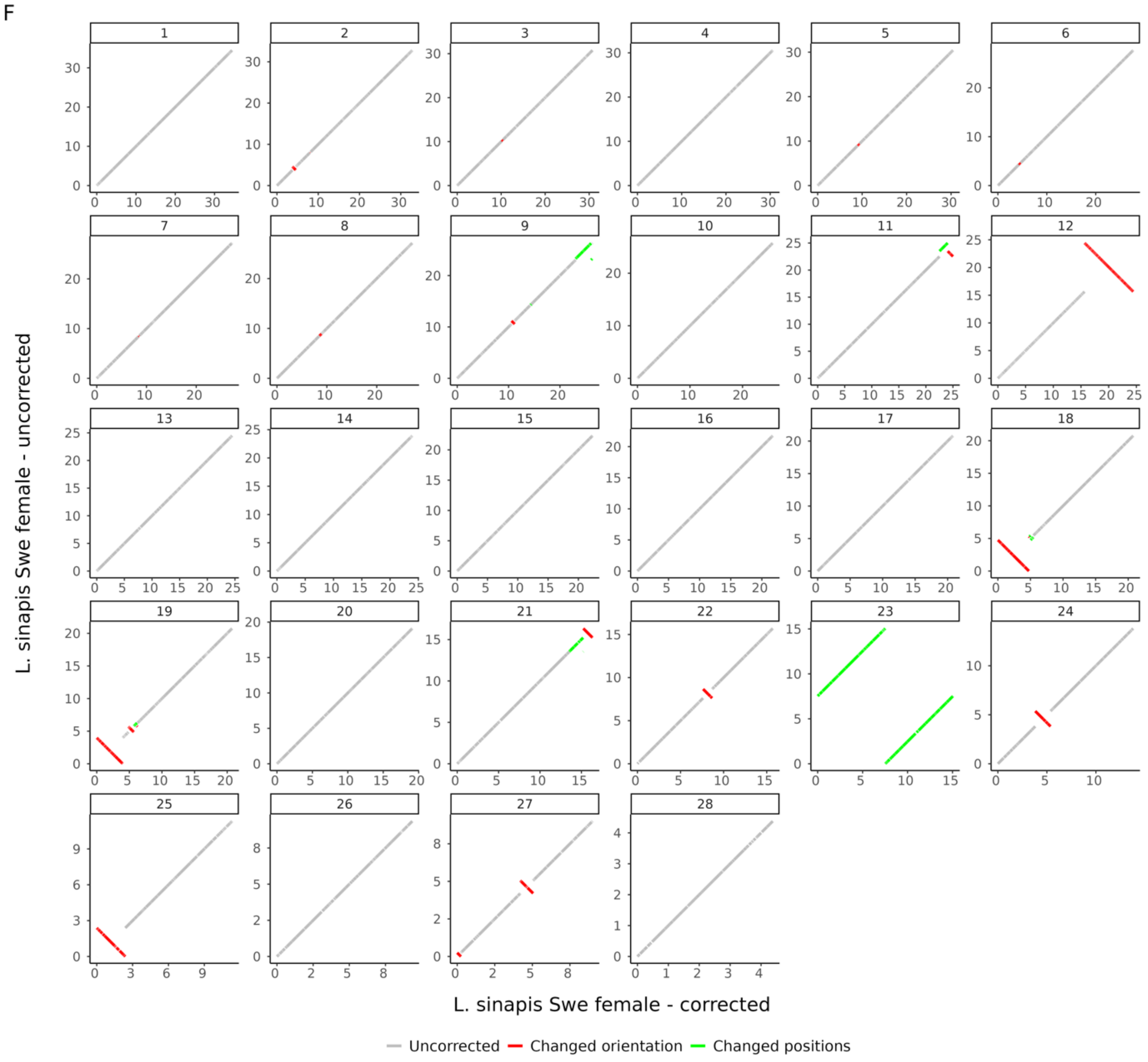

1
2
3
4
5
6
7
8
9
10
11
12
13
14
15
16
17
18
19
20
21
22
23
24
25
26
27
28
L. sinapis Swe female - uncorrected
L. sinapis Swe female - corrected
Uncorrected
Changed orientation
Changed positions

G

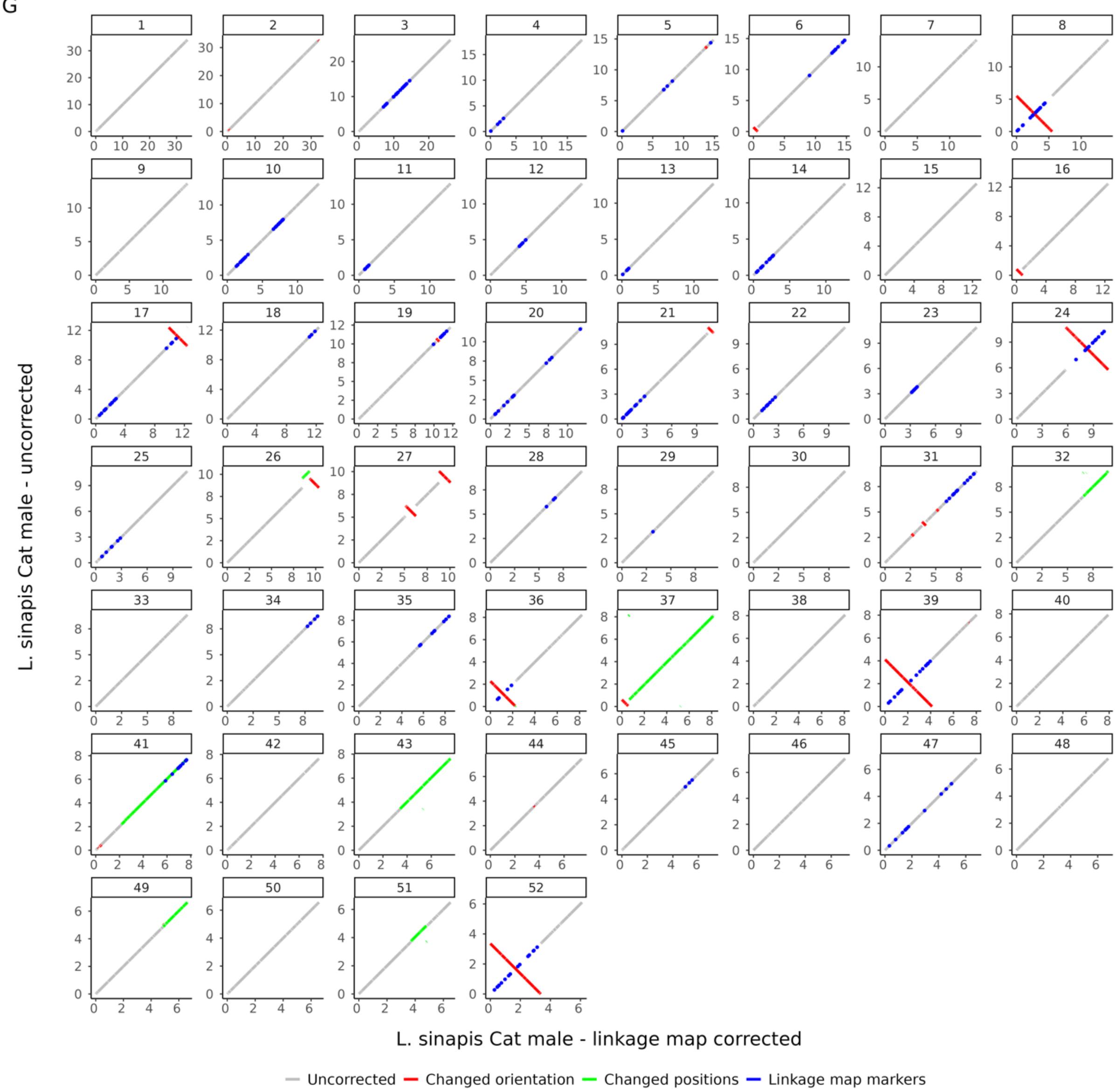
1 2 3 4 5 6 7 8 9 10 11 12 13 14 15 16 17 18 19 20 21 22 23 24 25 26 27 28 29 30 31 32 33 34 35 36 37 38 39 40 41 42 43 44 45 46 47 48 49 50 51 52
L. sinapis Cat male - uncorrected
L. sinapis Cat male - linkage map corrected
Uncorrected
Changed orientation
Changed positions
Linkage map markers

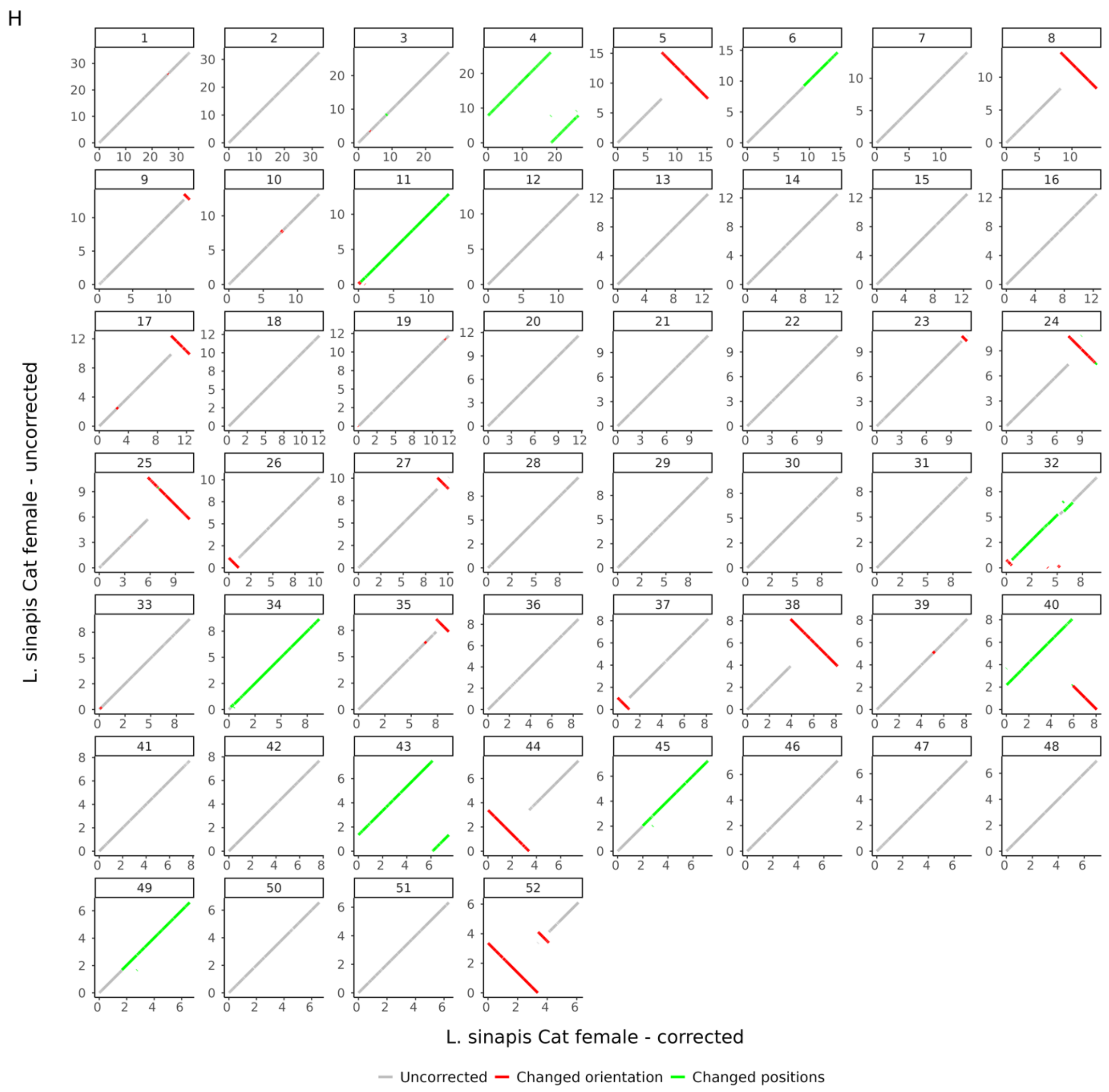

Supplementary figure 2. Sequence alignments comparing before (query) and after (reference) assembly corrections guided by linkage map information (E and M), HiC data and pairwise alignments (A-H) between species and populations. Linkage map markers (highlighted in blue) show markers deviating from the physical assembly positions. Scale is in mb. A: L. juvernica male, B: L. juvernica female, C: L. reali male, D: L. reali female E: L. sinapis Swe male, F: L. sinapis Cat male, G: L. sinapis Swe female, H: L. sinapis Cat female.

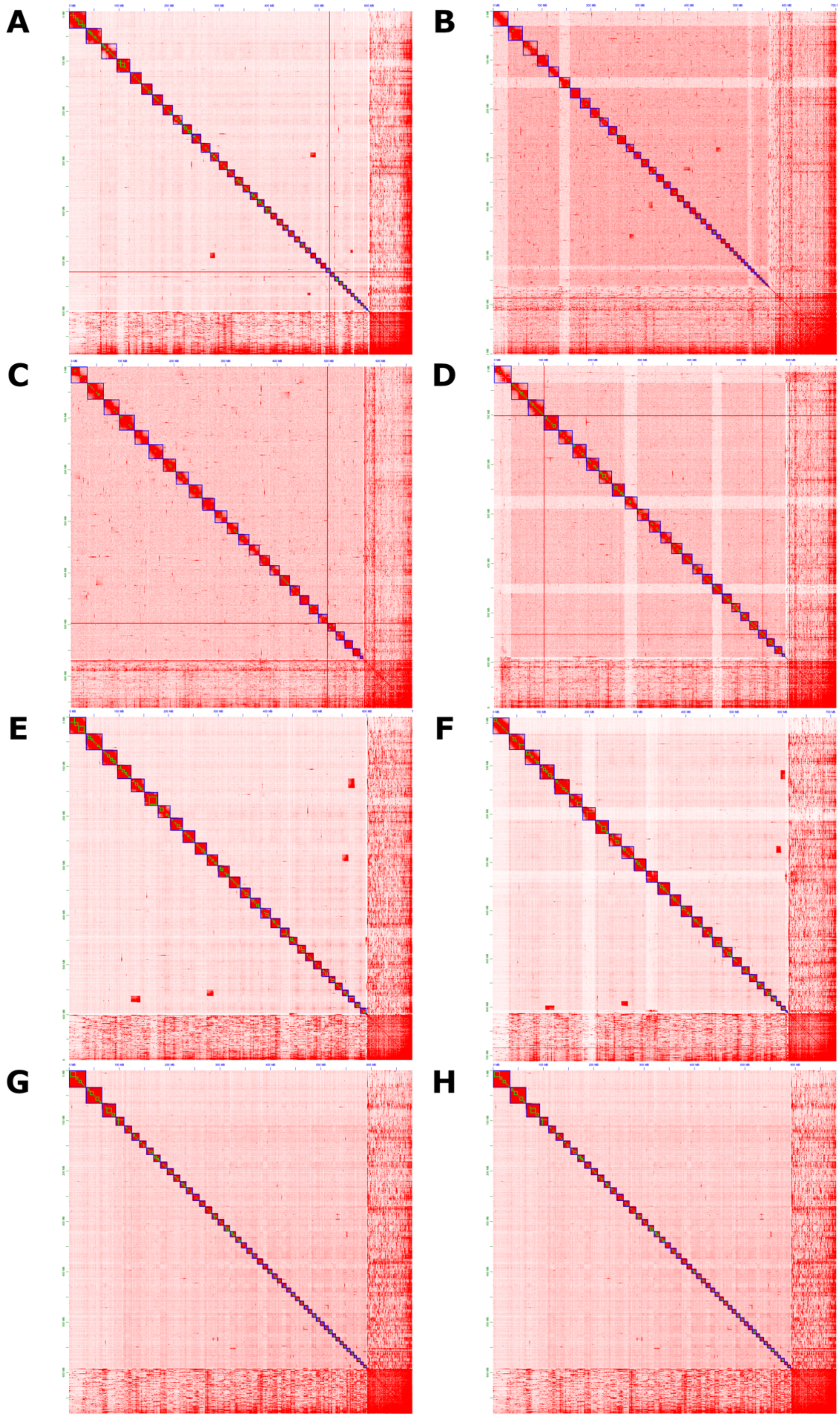

Supplementary figure 3. Heatmaps of HiC interactions across the genomes. A: L. juvernica male, B: L. juvernica female, C: L. reali male, D: L. reali female, E: L. sinapis Swe male, F: L. sinapis Swe female, G: L. sinapis Cat male, H: L. sinapis female.

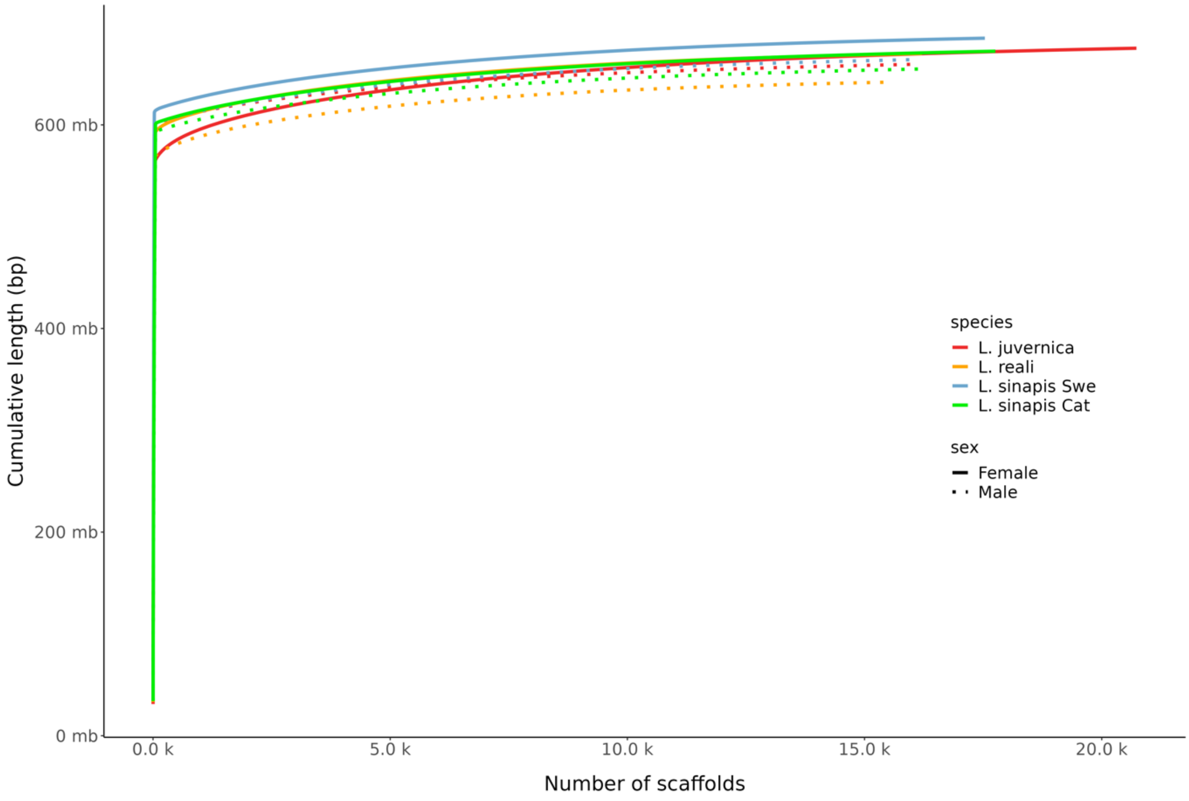

Supplementary figure 4. Cumulative scaffold length. Scaffolds are ordered by size.

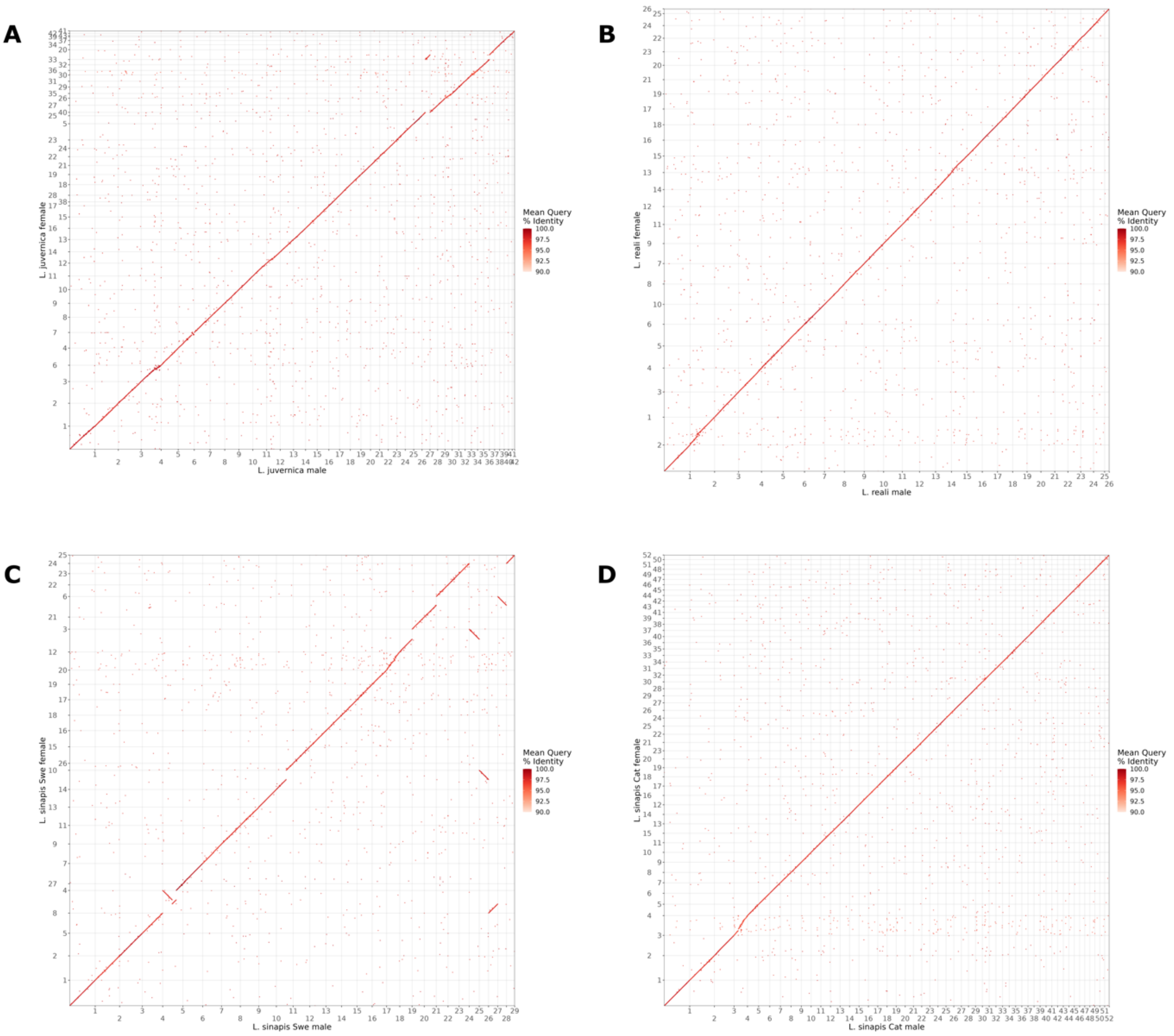

Supplementary figure 5. Dotplots of alignments (>1kb) between Leptidea male and female assemblies. Scaffolds in the query are oriented so the majority of alignments are in the same direction as the reference. Mean query % identity shows mean identity for all alignments in each scaffold. A: L. juvernica, B: L. reali, C: L. sinapis Swedish population and D: L. sinapis Catalan population.

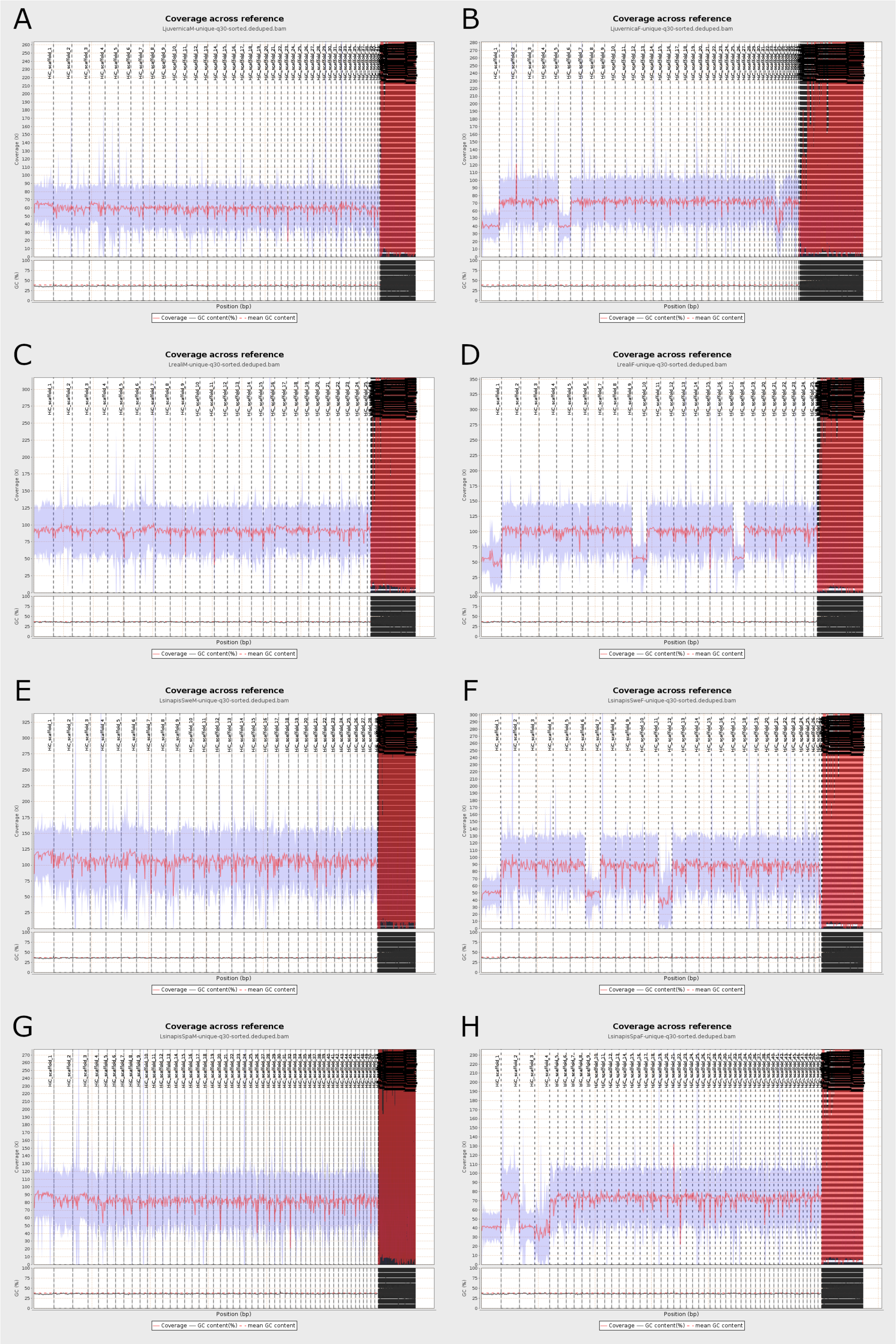

Supplementary figure 6. Read mapping coverage and GC% across scaffolds. The red line shows mean coverage in 600 windows across the genome and the blue area shows standard deviation. Vertical dotted lines show scaffold boundaries. A: L. juvernica male, B: L. juvernica female, C: L. reali male, D: L. reali female, E: L. sinapis Swe male, F: L. sinapis Swe female, G: L. sinapis Cat male, H: L. sinapis female.

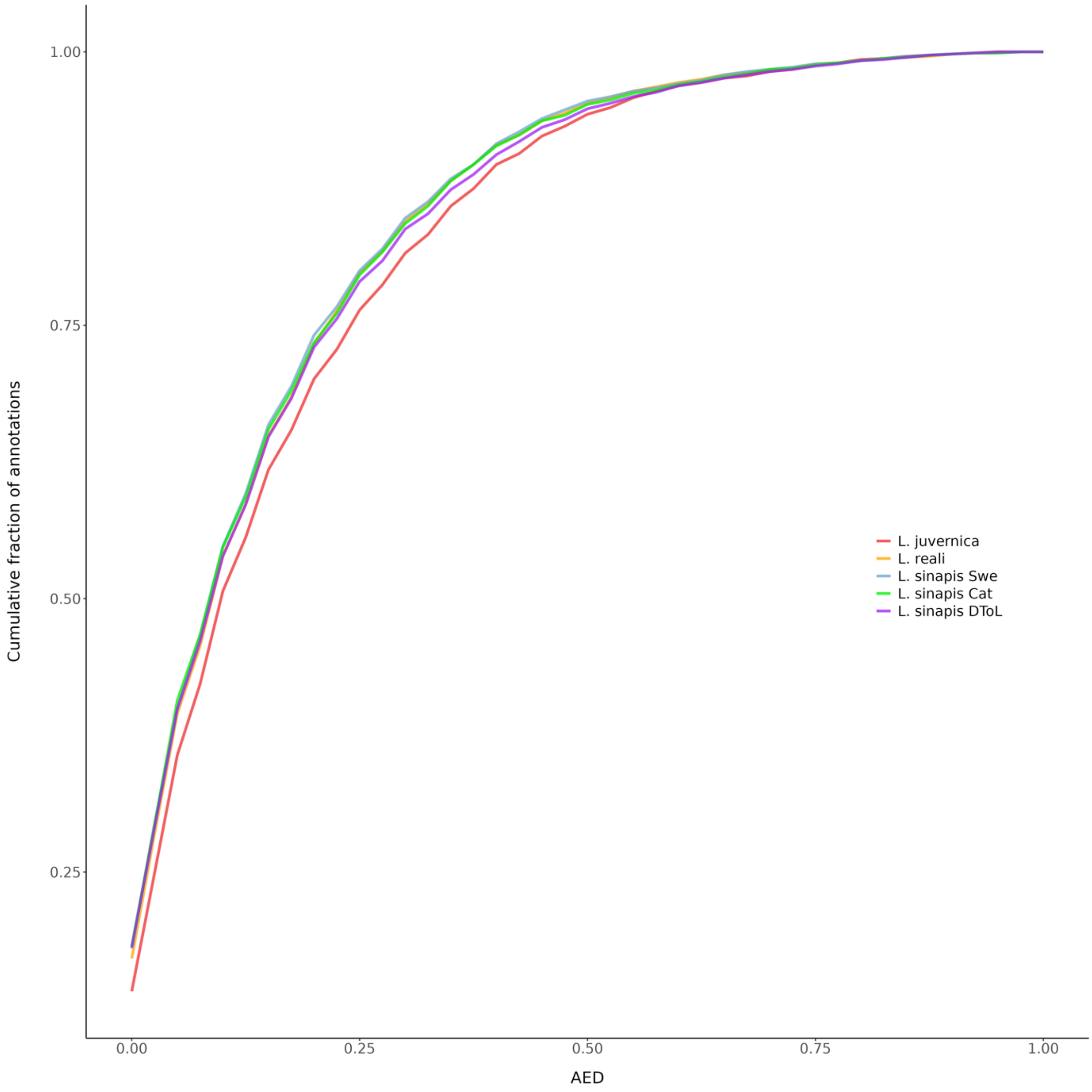

Supplementary figure 7. Cumulative AED score for gene annotations. A score of 0 indicates full congruence and 1 indicates lack of congruence between the evidence and the final annotation.

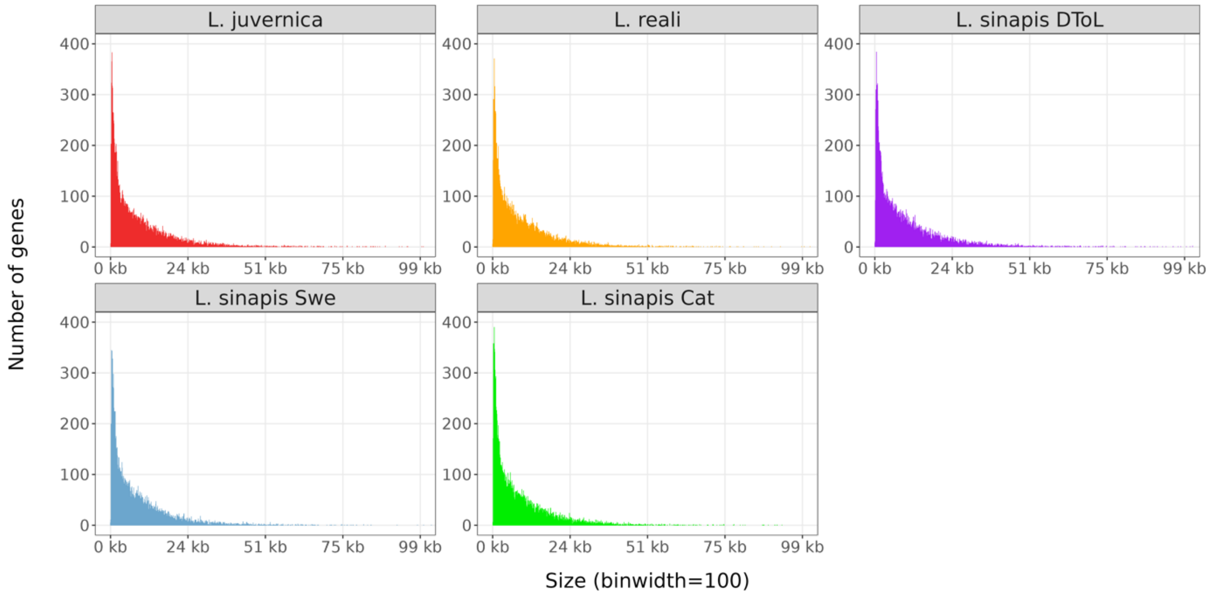

Supplementary figure 8. Histogram showing size (bp) distribution of predicted genes. The x-axis has been capped at 100kb.

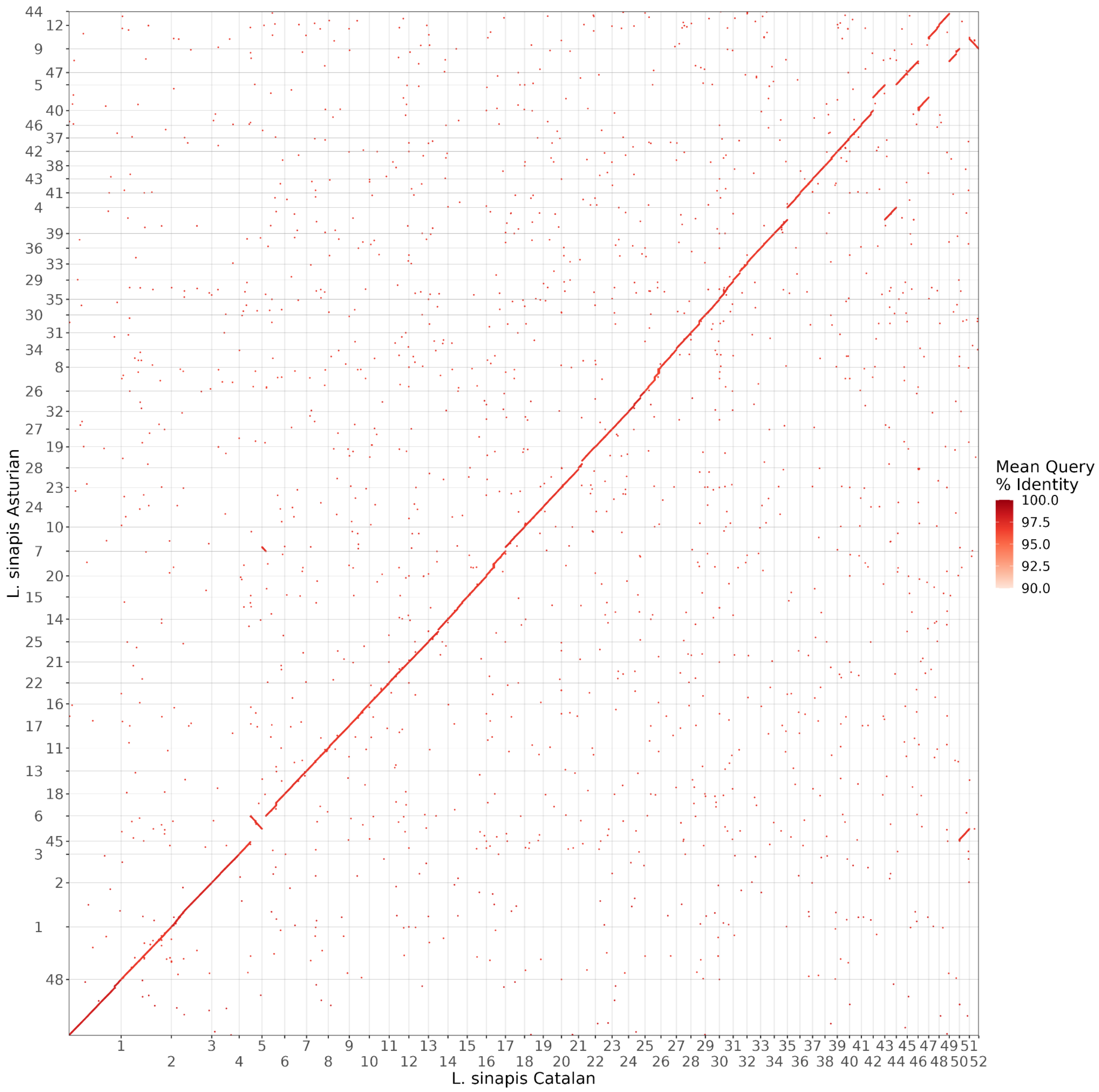

Supplementary figure 9. Dotplot of alignments (>1kb) between L. sinapis Catalan male and L. sinapis Asturian (DToL) assemblies. Scaffolds in the query are oriented so the majority of alignments are in the same direction as the reference. Mean query % identity shows mean identity for all alignments in each scaffold.

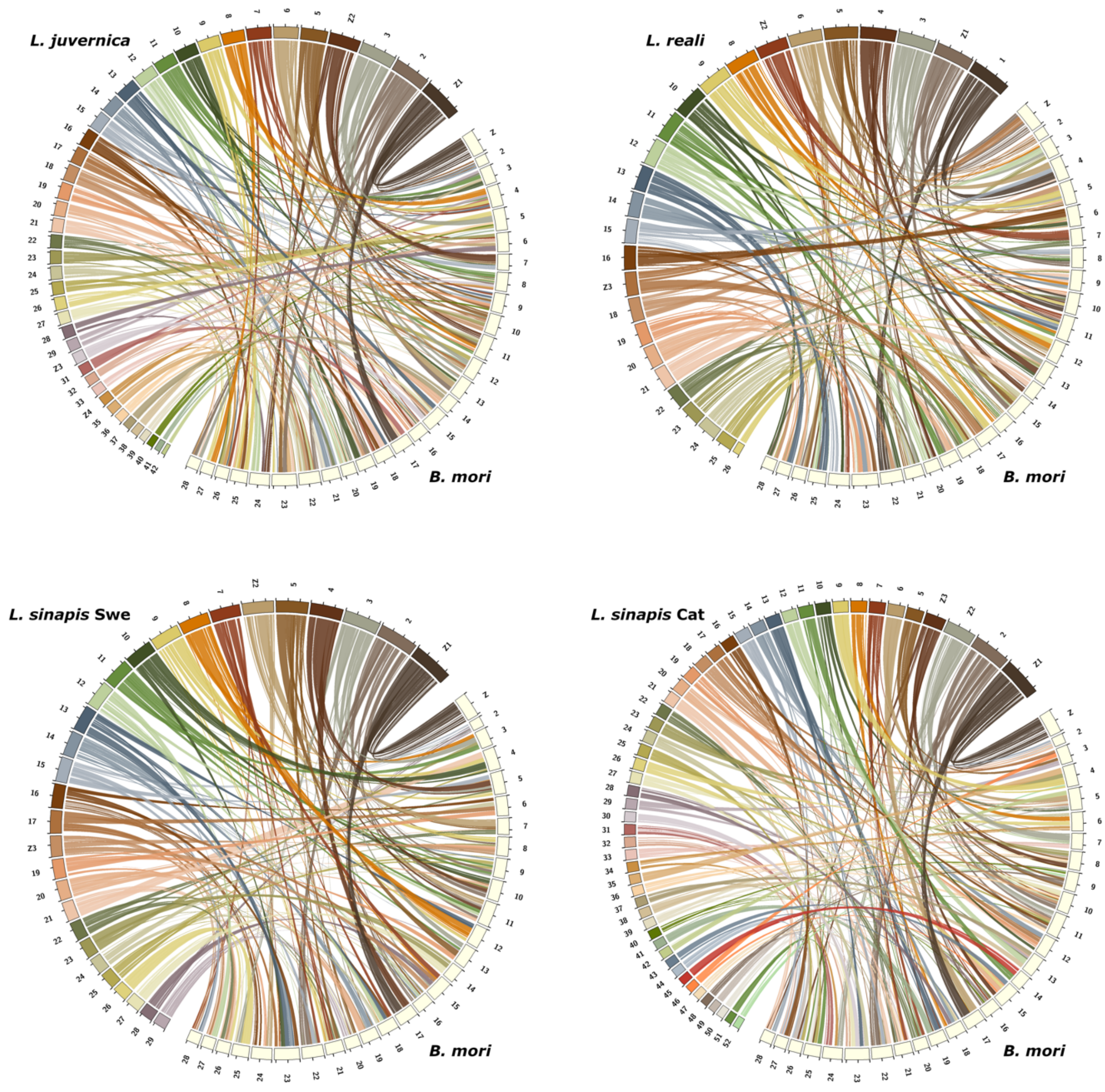

Supplementary figure 10. Syntenic relationship between Leptidea species and Bombyx mori.

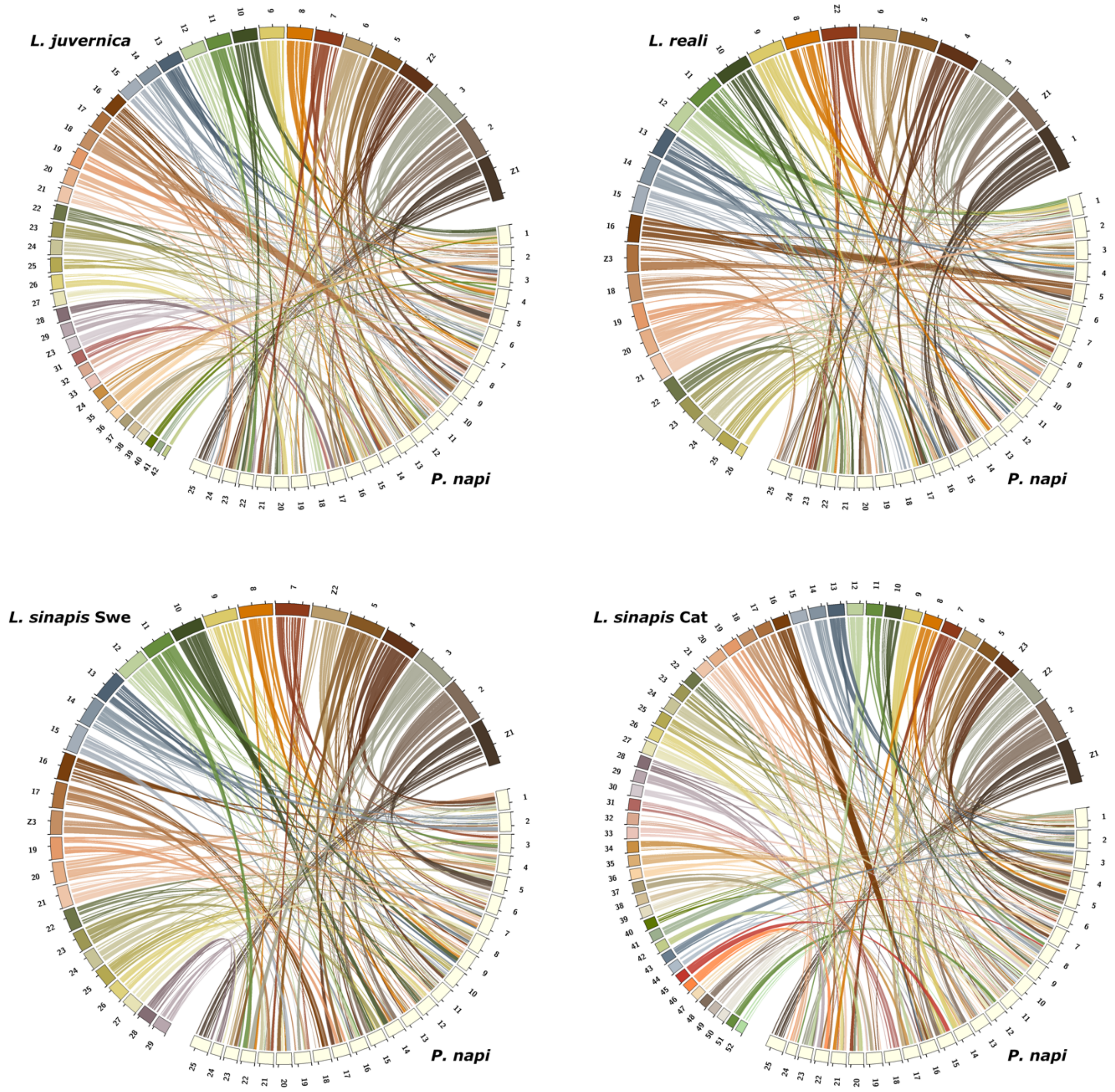

Supplementary figure 11. Syntenic relationship between Leptidea species and Pieris napi.

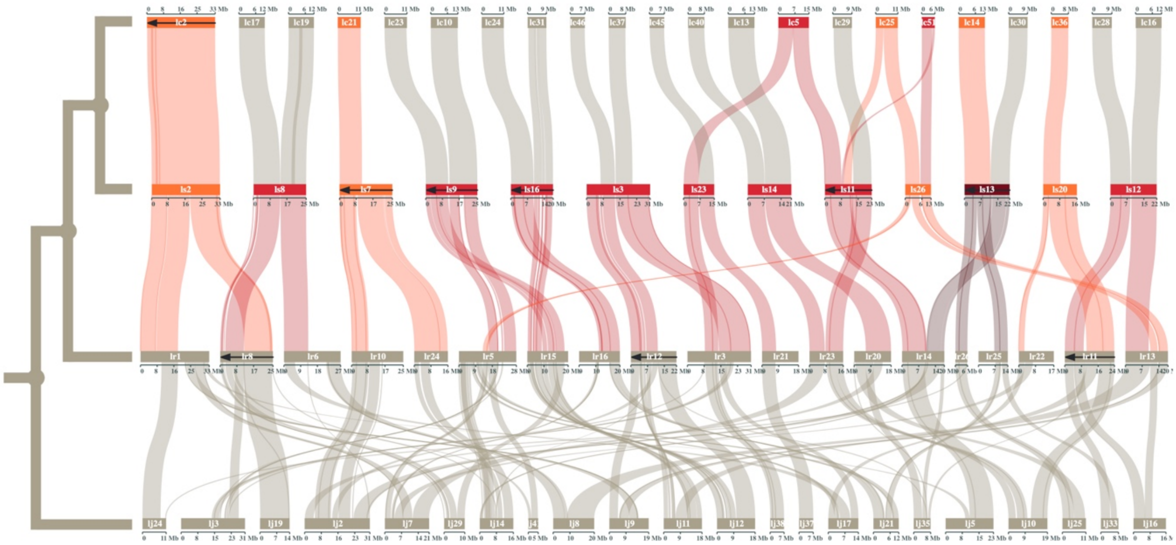

Supplementary figure 12. Inferred fusion events in L. sinapis. Unique fusions are highlighted in red and ancestral fusions are highlighted in orange. Darker colour show chromosomes resulting from two events. Chromosomes with arrows have been rotated to simplify visualization. lc: L. sinapis Catalan karyotype, ls: L. sinapis Swedish karyotype, lr: L. reali and lj: L. juvernica.

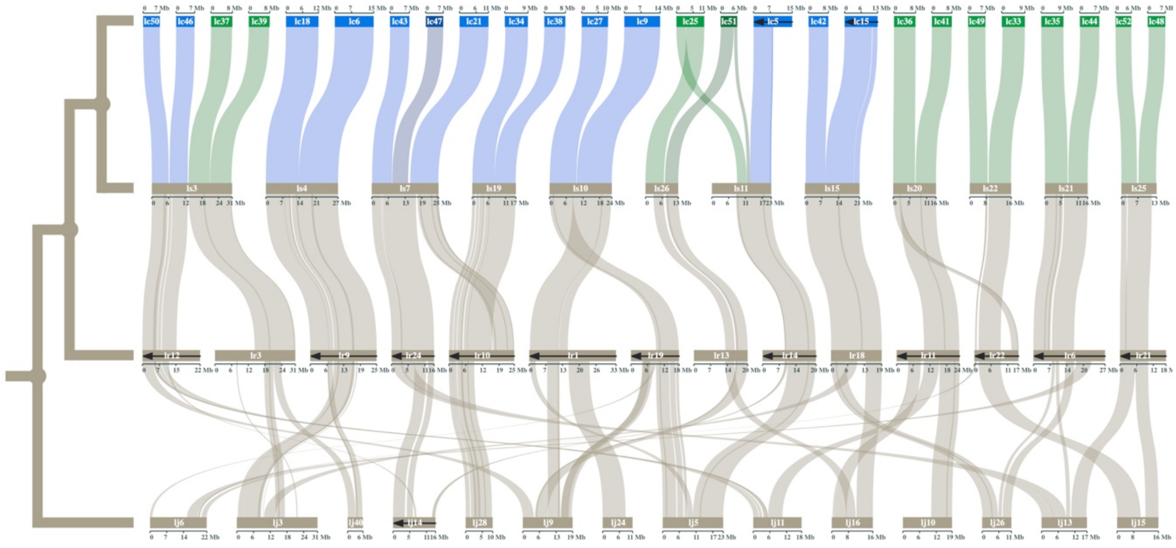

Supplementary figure 13. Inferred fission events in L. sinapis. Unique fissions are highlighted in blue and fissions shared between L. sinapis Catalan karyotype and L. juvernica are highlighted in green. Darker colour show chromosomes resulting from two events. Chromosomes with arrows have been rotated to simplify visualization. lc: L. sinapis Catalan karyotype, ls: L. sinapis Swedish karyotype, lr: L. reali and lj: L. juvernica.

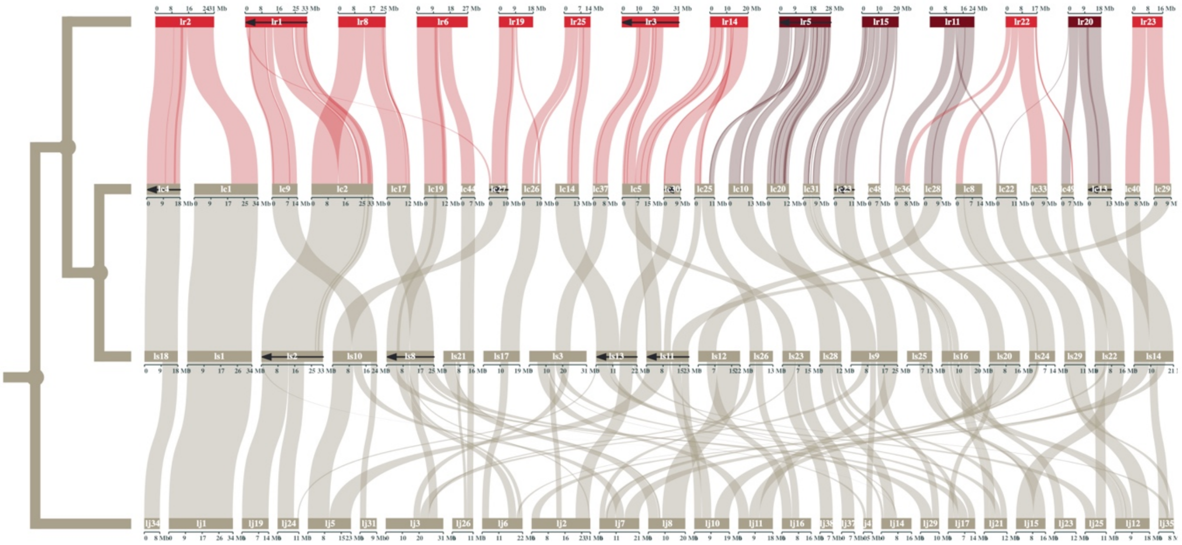

Supplementary figure 14. Inferred fusion events in L. reali. Fusions are highlighted in red. Darker colour show chromosomes resulting from two events. Chromosomes with arrows have been rotated to simplify visualization. lr: L. reali , lc: L. sinapis Catalan karyotype, ls: L. sinapis Swedish karyotype and lj: L. juvernica.

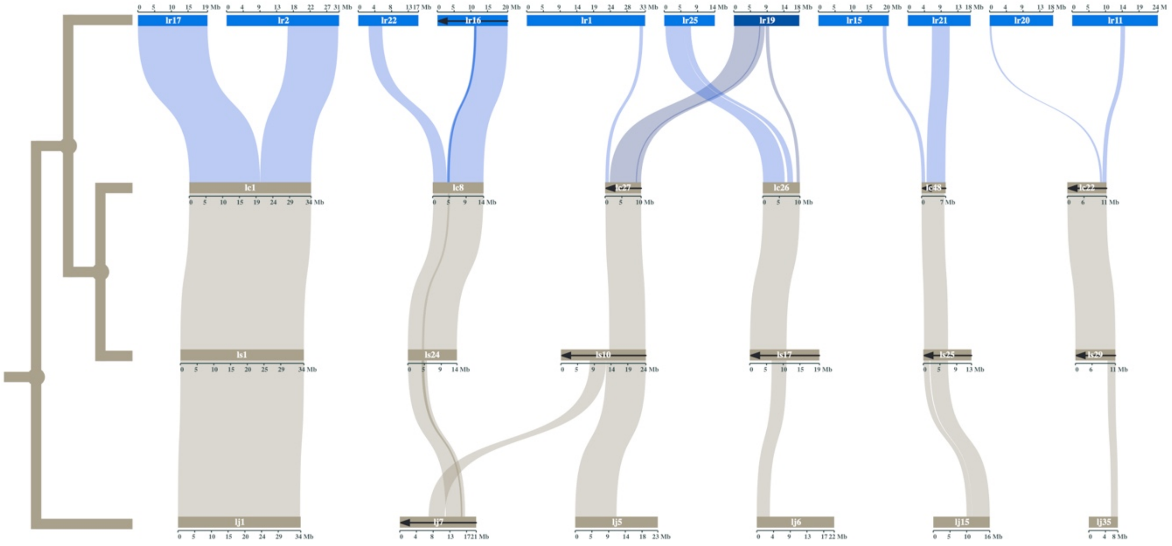

Supplementary figure 15. Inferred fission events in L. reali. Fissions are highlighted in blue. Darker colour show chromosomes resulting from two events. Chromosomes with arrows have been rotated to simplify visualization. lr: L. reali, lc: L. sinapis Catalan karyotype, ls: L. sinapis Swedish karyotype and lj: L. juvernica.

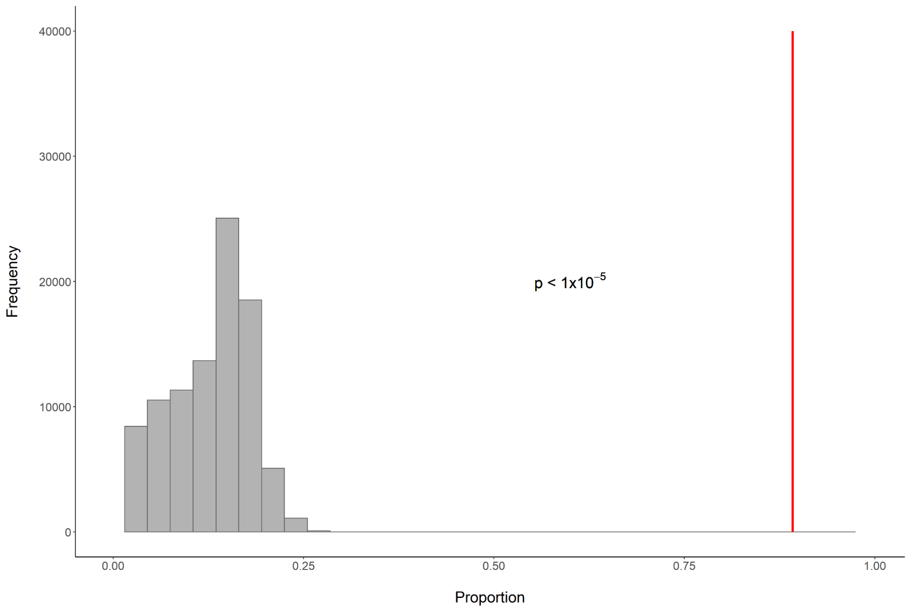

Supplementary figure 16. Comparison of the observed proportion of identified fusions in Leptidea with overlapping breakpoints against M. cinxia to a distribution of random overlaps with breakpoints in Leptidea against M. cinxia. The distribution was generated by random sampling of non-overlapping windows across the combined Leptidea genomes and counting the proportion of windows that overlapped a breakpoint identified between Leptidea and M. cinxia. Each sample contained the equivalent number and sizes as all called breakpoints between Leptidea species and the sampling was repeated 100k times. The red line shows the observed proportion of called fusions that overlapped with breakpoints between Leptidea and M. cinxia. The p-value indicates the probability of observing the proportion given the distribution and was calculated with a one-tailed significance test.

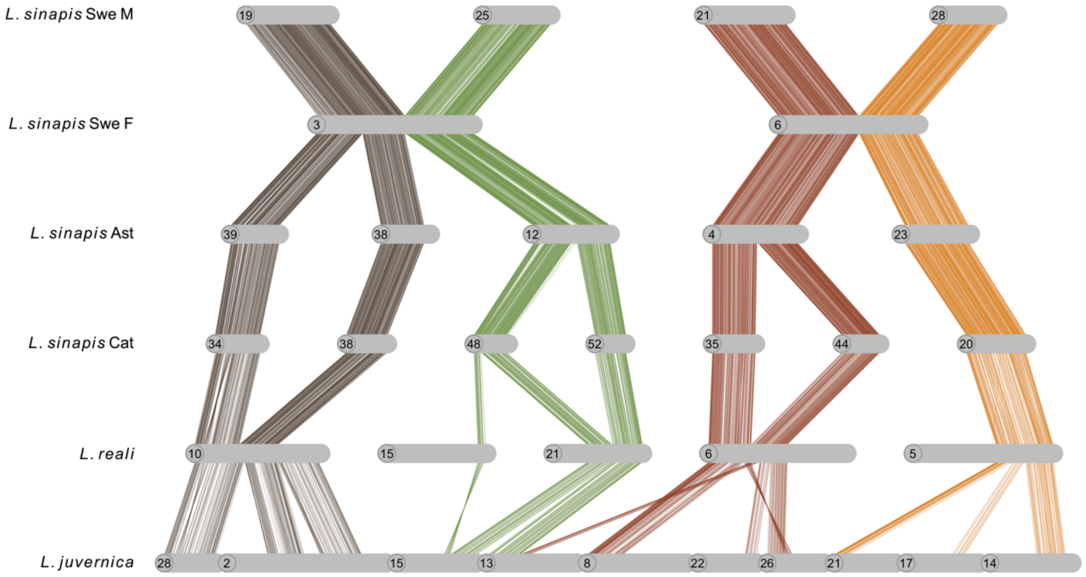

Supplementary figure 17. Segregating chromosome fusions in the Swedish L. sinapis population identified in the genome assemblies. The two fusions were unique for the female individual. Chromosomes have been rotated to enhance visualization. Ast: Asturias population, Cat: Catalan population, Swe: Swedish population.

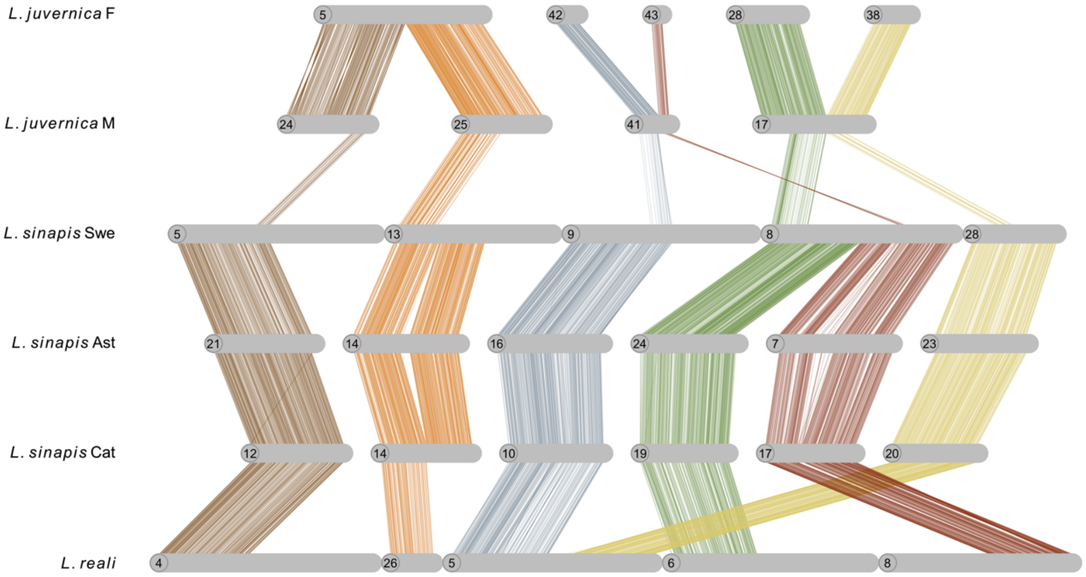

Supplementary figure 18. Segregating chromosome fusions in L. juvernica. Lines show individual alignments (> 90% similarity) and colours represent homologous regions. Chromosomes have been rotated to enhance visualization. Ast: Asturias population, Cat: Catalan population, Swe: Swedish population.

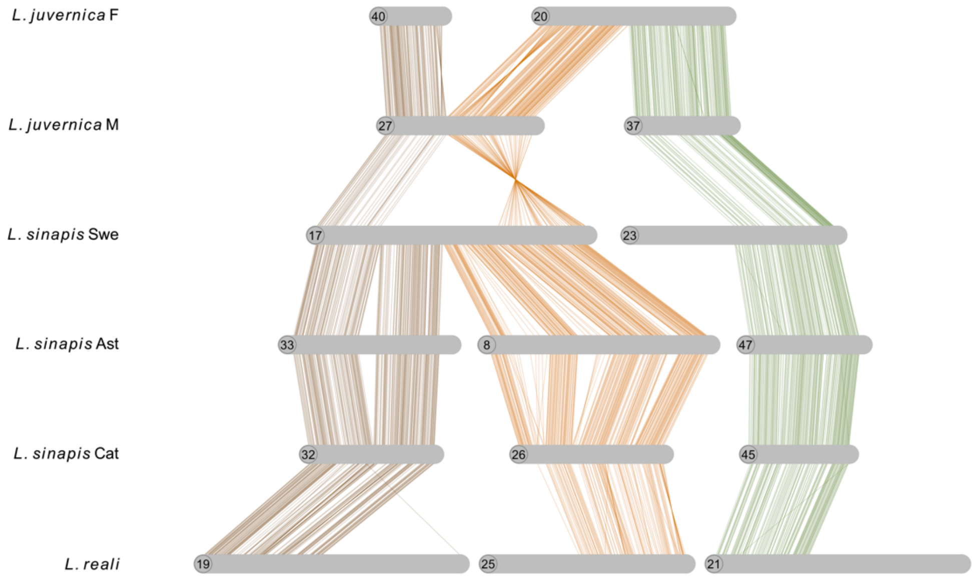

Supplementary figure 19. Rearrangement polymorphism in L. juvernica. Lines show individual alignments (> 90% similarity) and colours represent homologous regions. Chromosomes have been rotated to enhance visualization. Ast: Asturias population, Cat: Catalan population, Swe: Swedish population.

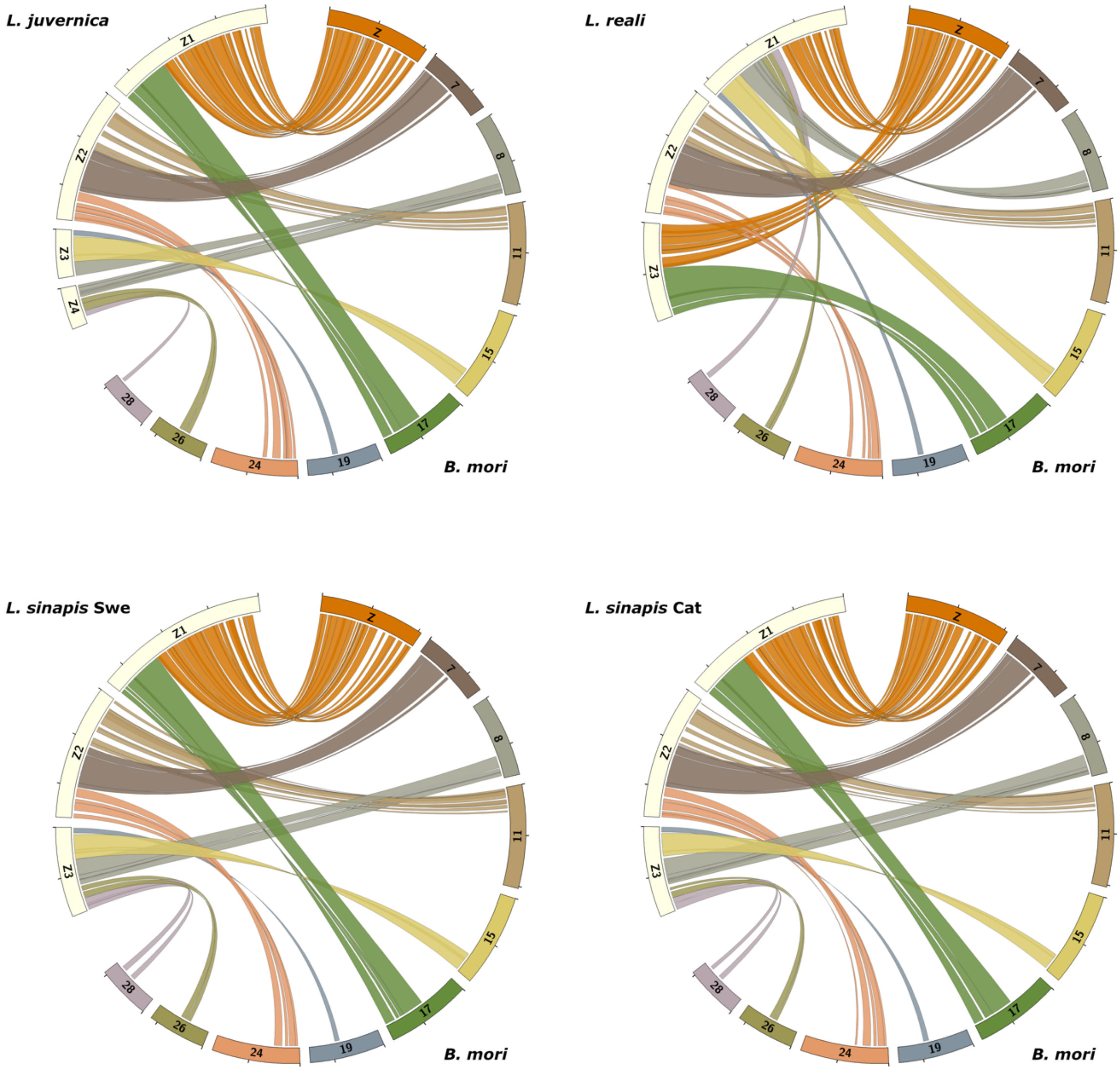

Supplementary figure 20. Synteny between Leptidea sex chromosomes and homologous B. mori chromosomes. Individual Leptidea chromosomes have been rotated to simplify comparison between species.

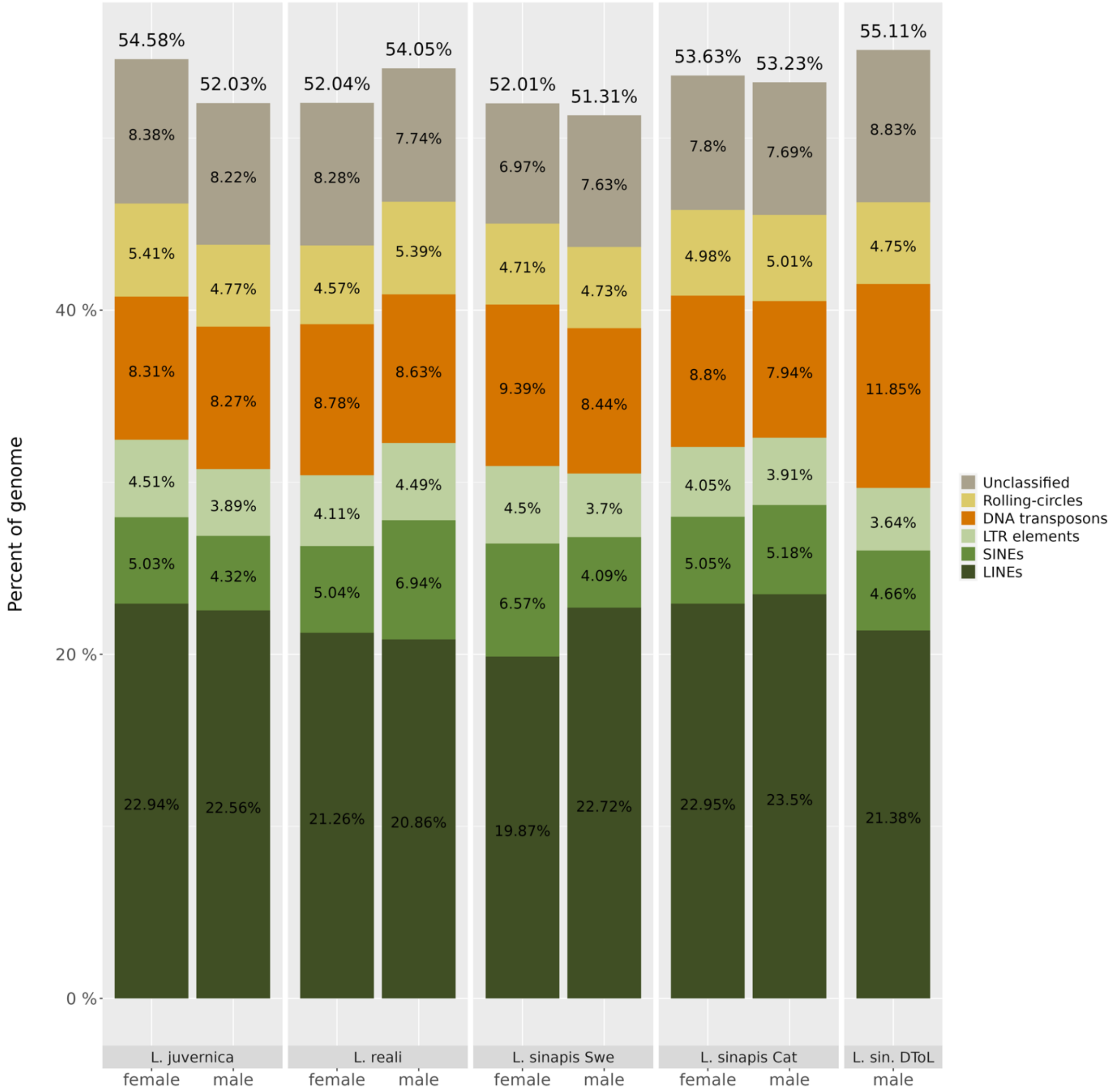

Supplementary figure 21. Stacked barplot showing percent of different types of transposable element in the Leptidea genome assemblies. Percent values show assembly sequence content per repeat class and values on top of each bar shows total TE content. LINE = long interspersed elements, SINE = short interspersed elements, LTR = long terminal repeats.

Supplementary tables

Supplementary table 1. BUSCO scores in %, showing the estimated completeness of core Lepidoptera genes. The database used was lepidoptera_odb10 containing 5 286 genes. Values in parentheses show percent unit changes from uncorrected and unfiltered assemblies.

Assembly	Complete (C)	Complete and single-copy (S)	Complete and duplicated (D)	Fragmented (F)	Missing (M)
L. juvernica male	96.9% (-0.3%)	95.8% (+0.1%)	1.1% (-0.4%)	1.1%	2.0% (+0.3%)
L. juvernica female	95.0% (-0.1%)	93.5%	1.5% (-0.1%)	2.2	2.8% (+0.1%)
L. reali male	95.1% (-0.2%)	94.1% (+0.1%)	1.0% (-0.3%)	2.0%	2.9% (+0.2%)
L. reali female	96.2% (-0.2%)	94.2% (+0.1%)	1.9% (-0.3%)	1.5%	2.3% (+0.2%)
L. sinapis Swe male	96.0% (-0.1%)	94.7 (+0.4)%	1.3% (-0.5%)	1.4 (-0.1)%	2.6% (+0.2%)
L. sinapis Swe female	96.3% (-0.1%)	94.6% (+0.1)%	1.7% (-0.2)%	1.5%	2.2% (+0.1%)
L. sinapis Cat male	96.3% (-0.3%)	95.2%	1.1% (-0.3%)	1.2%	2.5% (-0.3%)
L. sinapis Cat female	96.7% (-0.1%)	95.1% (+0.1%)	1.6% (-0.2%)	1.2%	2.1% (+0.1%)

Supplementary table 2. Female identity and number of offspring per female (# offspring) used in the pedigree for constructing linkage maps in the Swedish and Catalonian *L. sinapis* populations.

Swedish		Catalonian	
Female	# offspring	Female	# offspring
T2	41	3c9	38
T3	31	4C	35
T4	30	7C	34
T5	58	7CB	20
T6	14	8C	27
S25	10	9C	24
Total	184	Total	178

Supplementary table 3. Sex chromosome identification and statistics. Note that Z chromosomes with similar chromosome names are not necessarily homologous between species.

Assembly	Chromosome name	Scaffold name	Size (mb)	Chr:genome mean read depth ratio
L. juvernica male	Z1	HiC_scaffold_1	34.1	1.0533
	Z2	HiC_scaffold_4	26.8	1.0216
	Z3	HiC_scaffold_30	9.7	0.9931
	Z4	HiC_scaffold_34	8.3	1.0031
L. juvernica female	Z1	HiC_scaffold_1	31.2	0.5847
	Z2	HiC_scaffold_6	22.0	0.5793
	Z3	HiC_scaffold_35	6.4	0.5816
	Z4	HiC_scaffold_36	6.3	0.7444
L. reali male	Z1	HiC_scaffold_2	31.4	1.0356
	Z2	HiC_scaffold_7	25.4	1.0480
	Z3	HiC_scaffold_17	19.4	1.0585
L. reali female	Z1/W	HiC_scaffold_1	35.4	0.5650
	Z2	HiC_scaffold_10	25.4	0.5988
	Z3	HiC_scaffold_18	19.6	0.6036
L. sinapis Swe male	Z1	HiC_scaffold_1	34.5	1.1004
	Z2	HiC_scaffold_6	26.6	1.0702
	Z3	HiC_scaffold_18	17.8	1.0444
L. sinapis Swe female	Z1	HiC_scaffold_1	34.5	0.6211
	Z2	HiC_scaffold_7	27.2	0.6058
	Z3/W	HiC_scaffold_12	24.4	0.5066
	W	HiC_scaffold_28	4.4	0.4936
L. sinapis Cat male	Z1	HiC_scaffold_1	34.0	1.1052
	Z2	HiC_scaffold_3	26.2	1.0653
	Z3	HiC_scaffold_4	17.9	1.0207
L. sinapis Cat female	Z1	HiC_scaffold_1	34.2	0.6146
	Z2	HiC_scaffold_3	26.9	0.6140
	Z3/W	HiC_scaffold_4	26.3	0.5441

Supplementary table 4. Gene annotation statistics.

Assembly	Predicted genes (N)	Median gene size (bp)	Median CDS size (bp)	Median number of exons (N)	Median exon size (bp)	Genes on chromosome scaffolds (%)	Genes with functional predictions (N)	Predicted protein domains (N)
L. juvernica	16 149	5 444	789	4	148	92.41%	9 441	22 676
L. reali	15 689	5702	768	4	144	87.51%	9 256	21 452
L. sinapis Swe	15 915	5 453	762	4	144	90.34%	9 348	21 790
L. sinapis Cat	16 478	5 375	762	4	145	91.69%	9 723	22 724
L. sinapis DToL	17 229	6018	819	4	145	100%	9 935	23 684

Supplementary table 5. Synteny statistics from comparison between *Leptidea* species and *B. mori*

Statistic	*L. juvernica*	*L. reali*	*L. sinapis* Swe	*L. sinapis* Cat
Synteny blocks (N)	410	398	372	390
Median synteny blocks / chr	14.00	14.50	13.00	12.50
Median *B. mori* chr / chr	4.00	5.00	5.00	3.00
Median block size (mb)	0.96	0.94	1.00	0.96
Total genes in synteny blocks (N)	13 998	12 912	13 308	13 914
Median genes / block	25.00	24.00	24.00	24.00
Total orthologs in synteny blocks (N)	7 894	6 947	7 405	7 840
Median orthologs / block	12.00	11.00	12.00	13.00

Supplementary table 6. Inferred fission and fusion events in *Leptidea*. Shaded chromosomes represent the reference for each inference. Columns with fusion events show the number of events for each respective reference chromosome and query chromosomes involved in fusions are in bold. Columns with fission events show which query chromosomes that result from an event. Values/chromosomes in parentheses show calls that change polarity after comparison with *M. cinxia* and detection of telomeric repeats. Direction of manual corrections are indicated by the last column.

Chromosome number and homologous chromosomes in the other species				Fusion	Fusion anc *L. sin*	Fission *L. sin* Cat	Fission *L. rea*	Fission *L.juv* / *L. sin* Cat	Manual Correction
L. juvernica	*L. reali*	*L. sinapis* Swe	*L. sinapis* Cat						
1	2, 17	1	1				2-17		
2	6, 10, 12, 14, 20	3, 7, 8, 13, 14, 19	13, 19, 21, 30, 38, 46						
3	3, 4, 5, 8, 9, 18	3, 4, 5, 8, 9, 15	6, 7, 10, 15, 17, 18, 37, 42			6-18, 15-42			
4	7	6	3						
5	1, 14, 19	10, 11	5, 9, 25, 27, 51			(5-51), 9-27	1-19		
6	4, 6, 19, 22, 25	5, 8, 17, 22	12, 19, 26, 49				19-25		
7	1, 5, 16, 22, 23	10, 14, 24, 26	8, 9, 25, 40				16-22		
8	6, 13	12, 21	16, 35						
9	1, 12, 13, 19	3, 10, 17, 26	9, 32, 50, 51						
10	4, 11, 23	5, 11, 20	7, 29, 41						
11	11, 12, 15	3, 9, 20	23, 36, 46, 50			46-50			
12	12, 15	16, 29	22, 31						
13	6, 21, 24	7, 21, 25	43, 44, 47, 52			43-47			
14	5, 12, 14, 24	3, 7, 11, 28	20, 21, 25, 46, 47			21-47			
15	15, 21, 22	22, 25	33, 48				15-21		
16	11, 13, 18	12, 15, 26	15, 25, 28						
17	3, 5, 6, 22	3, 8, 20, 28	19, 20, 36, 37						
18	1, 20	2, 14	2, 13						
19	8	2	2						
20	4	27	11						
21	5, 16, 25	13, 16, 28	14, 20, 24						
22	3, 6	5, 21	7, 44						
23	9, 16	4, 16	18, 24						
24	1, 4	2, 5	2, 12						
25	20, 26	13, 22	14, 49						
26	6, 18	15, 21	35, 42, 44						
27	19, 25	17	26, 32						
28	10	19	34, 38			34-38			
29	8, 15	8, 9	17, 23						
30	2	18	4						
31	1, 9	4, 10	6, 9						
32	16	24	8						
33	4, 11, 25	5, 12, 13	7, 14, 28						
34	2	18	4						

35	11, 14, 20	13, 14, 29	13, 22, 30				11-20		
36	3, 9	3, 4	6, 39						
37	21	23	45						
38	3	23	5						
39	5	9	10						
40	3, 9	3, 4	18, 39						
41	5, 8	8, 9	10, 17						
42	18	15	15						
5, 7, 9, 18, **24**, **31**	1	**2**, **10**	**2**, **9**, 27	1		9-27			
**1**, 30, **34**	2	**1**, **18**	**1**, **4**	1					
3, **17**, 22, 36, **38**, 40	3	**3**, 5, **23**	**5**, 7, **37**, 39	1				1	
3, 6, 10, 20, 24, 33	4	5, 27	7, 11, 12					(1)	Fission -> Fusion
3, **7**, 14, 17, **21**, 39, **41**	5	**9**, **26**, **28**	**10**, **20**, **25**	2					
**2**, 6, 8, 13, 17, 22, **26**	6	**8**, **21**	**19**, 35, **44**	1				1	
4	7	6	3						
**3**, **19**, 29, 41	8	**2**, **8**	**2**, **17**	1					
3, 23, 31, 36, 40	9	4	6, 18			6-18			
2, 28	10	7, 19	21, 34, 38			34-38			
10, **11**, **16**, 33, **35**	11	**12**, **20**, **29**	**22**, **28**, **36**, 41	2				1	
2, 9, 11, 12, 14	12	3, 29	22, 46, 50			46-50			
**8**, 9, **16**	13	**12**, **26**	**16**, **25**, 51	(1)				1	Fusion -> Fission
**2**, 5, **14**, 35	14	**11**, **13**	5, **25**, **30**, 51	1		5-51			
**11**, **12**, **15**, **29**	15	**9**, **16**, **25**	**23**, **31**, **48**	2					
**7**, 21, **23**, 32	16	**16**, **24**	**8**, **24**	(1)					Fusion -> Fission
1	17	1	1						
3, 16, 26, 42	18	15	15, 42			15-42			
**5**, **6**, 9, 27	19	**10**, **17**	**26**, **27**, 32	1				(1)	Fission -> Fusion
2, 18, **25**, **35**	20	**14**, **22**, **29**	**13**, **22**, **49**	2					
13, **15**, **37**	21	**23**, **25**	**45**, **48**, 52	(1)				1	Fusion -> Fission
6, **7**, **15**, 17	22	20, **22**, **24**	**8**, **33**, 36, 49	1				1	
**7**, **10**	23	**11**, **14**	**29**, **40**	1					
13, 14	24	7	21, 43, 47			43-47, 21-47			
**6**, **21**, 27, 33	25	**13**, **17**	**14**, **26**	1					
25	26	13	14						
1	2, 17	1	1				2 -17		
18, **19**, **24**	**1**, **8**	2	2		1				
**2**, 3, 9, 11, 14, **17**, 36, 40	**3**, **12**	3	**37**, 39, **46**, 50	1		46-50		1	
3, 23, 31, 36, 40	9	4	6,18			6-18			
3, 6, 10, 22, 24, 33	3, 4	5	7,12					(1)	Fission -> Fusion

4	7	6	3						
**2**, 13, **14**	**10**, **24**	7	21, 43, 47		1	21-47, 43-47			
**2**, **3**, 6, 17, 29, 41	**6**, **8**	8	**17**, **19**	1					
3, 11, **29**, 39, **41**	**5**, **15**	9	**10**, **23**	1					
5, 7, 9, 31	1, 19	10	9, 27			9-27	1-19		
5, **10**, **14**	**14**, **23**	11	5, **25**, **29**, 51	1		5-51			
**8**, **16**, 33	**11**, **13**	12	**16**, **28**	1					
**2**, **21**, **25**, **33**, 35	**14**, **25**, **26**	13	**14**, **30**	1	1				
2, **7**, 18, **35**	**20**, **23**	14	**13**, **40**	1					
3, 16, 26, 42	18	15	15, 42			15-42			
**12**, **21**, 23	**15**, **16**	16	**24**, **31**	1					
6, 9, 27	19, 25	17	26, 32				19-25	(1)	Fission -> Fusion
30, 34	2	18	4						
2, 28	10	19	34, 38			34-38			
10, **11**, **17**	**11**, **22**	20	36, 41		1			1	
8, 13, 22, 26	6	21	35, 44					1	
6, 15, 25	20, 22	22	33, 49					1	
**37**, **38**	**3**, **21**	23	**5**, **45**	1					
7, 32	16, 22	24	8				16-22		
13, 15	15, 21	25	48, 52				15-21	1	
**7**, 9, **16**	**5**, **13**	26	25, 51		1			1	
20	4	27	11						
14, 17, 21	5	28	20						
12, 35	11, 12, 20	29	22				11-20		
1	2, 17	1	1				2-17		
18, **19**, **24**	**1**, **8**	2	2		1				
4	7	6	3						
30, 34	2	18	4						
**5**, **38**	**3**, **14**	**11**, **23**	5	1					
3, 31, 36	9	4	6						
3, 10, 22, 33	3, 4	5	7						
7, 32	16, 22	24	8				16-22		
5, 7, 9, 31	1	10	9						
3, 39, 41	5	9	10						
20	4	27	11						
6, 24	4	5	12						
2, 18, 35	20	14	13						
**21**, **25**, 33	**25**, **26**	13	14		1				
3, 16, 42	18	15	15						
8	13	12	16						
3, 29, 41	8	8	17						
3, 23, 40	9	4	18						
2, 6, 17	6	8	19						
14, 17, 21	5	28	20						

**2**, **14**	**10**, **24**	7	21		1				
12, 35	11, 12, 20	29	22				11-20		
11, 29	15	9	23						
21, 23	16	16	24						
5, **7**, 14, **16**	**5**, **13**, 14	11, 26	25		1				
6, 27	19, 25	17	26				19-25		
5	1, 19	10	27				1-19		
16, 33	11	12	28						
10	23	11	29						
2, 35	14	13	30						
12	15	16	31						
9, 27	19	17	32						
15	22	22	33						
28	10	19	34						
8, 26	6	21	35						
**11**, **17**	**11**, **22**	20	36		1				
3, 17	3	3	37						
2, 28	10	19	38						
36, 40	3	3	39						
7	23	14	40						
10	11	20	41						
3, 26	18	15	42						
13	24	7	43						
13, 22, 26	6	21	44						
37	21	23	45						
2, 11, 14	12	3	46						
13, 14	24	7	47						
15	15, 21	25	48				15-21		
6, 25	20, 22	22	49						
9, 11	12	3	50						
**5**, **9**	**13**, **14**	**11**, **26**	51	1					
13	21	25	52						

Supplementary table 7. Summary statistics of genetic element densities in fusion and fission breakpoints. Densities are shown as the mean percent of a breakpoint type covered by each sequence element type. P-values were calculated using two-tailed significance tests and indicate the probability of observing the mean given resampling distributions taken from the rest of the genomes. CDS = coding sequence, LINE = long interspersed elements, SINE = short interspersed elements, LTR = long terminal repeats, DNA = dna transposons, RC = rolling-circle TEs.

Breakpoint	Inference type	Element	Mean (%)	Standard deviation (%)	FDR adjusted p-value	Raw p-value
fusion	reference	CDS	2.54	1.45	0.932	0.918
”	“	LINE	29.33	7.23	**< 2*10^-5**	< 2*10^-5
“	“	SINE	3.46	1.64	**< 2*10^-5**	< 2*10^-5
“	“	LTR	6.30	3.02	**< 2*10^-5**	< 2*10^-5
“	“	DNA	8.39	2.71	0.932	0.893
“	“	RC	4.15	2.26	**0.003**	0.001
“	query	CDS	2.71	2.81	0.252	0.126
“	“	LINE	28.80	10.05	**< 2*10^-5**	< 2*10^-5
“	“	SINE	3.63	3.07	**< 2*10^-5**	< 2*10^-5
“	“	LTR	5.16	4.15	0.096	0.044
“	“	DNA	7.95	3.93	0.096	0.043
“	“	RC	4.50	2.92	**< 2*10^-5**	< 2*10^-5
fission	reference	CDS	2.92	2.40	0.644	0.456
”	“	LINE	24.54	5.93	0.919	0.805
“	“	SINE	4.83	2.25	0.644	0.432
“	“	LTR	4.29	2.63	0.448	0.280
“	“	DNA	9.05	3.43	0.370	0.205
“	“	RC	5.21	2.33	0.853	0.711
“	query	CDS	4.40	5.34	0.077	0.029
“	“	LINE	24.24	7.15	0.853	0.699
“	“	SINE	5.18	2.44	0.932	0.932
“	“	LTR	4.65	3.15	0.216	0.370
“	“	DNA	9.05	4.01	0.758	0.569
“	“	RC	4.32	1.92	**0.040**	0.013

Supplementary table 8. LINE element families enriched in telomeric regions in the Asturian *L. sinapis* assembly, identified with Fisher's exact test. The table is limited to the top five candidate families which are defined by occurring in more than half of the chromosomes and at least the same number of copies as the number of telomeres (n=96).

LINE family	Odds ratio	FDR-adjusted p-value	Raw p-value
rnd-4_family-1088 LINE/R1	120.57	$< 1 \times 10^{-189}$	$< 1 \times 10^{-191}$
rnd-4_family-5536 LINE/R1	58.41	$< 1 \times 10^{-189}$	$< 1 \times 10^{-191}$
rnd-4_family-980 LINE/R1	33.93	1×10^{-189}	1×10^{-191}
rnd-5_family-614 LINE/R2	39.99	1×10^{-136}	1×10^{-138}
rnd-5_family-1424 LINE/I-Jockey	6.30	2×10^{-57}	2×10^{-59}
